# The sugar kelp *Saccharina latissima* I: recent advances in a changing climate

**DOI:** 10.1093/aob/mcad173

**Published:** 2023-12-18

**Authors:** Nora Diehl, Huiru Li, Lydia Scheschonk, Bertille Burgunter-Delamare, Sarina Niedzwiedz, Silje Forbord, Maren Sæther, Kai Bischof, Catia Monteiro

**Affiliations:** Marine Botany, Faculty of Biology and Chemistry, University of Bremen, 28359 Bremen, Germany; Key Laboratory of Mariculture (Ministry of Education), Fisheries College, Ocean University of China, Qingdao 266003, China; Independent Researcher; Matthias Schleiden Institute of Genetics, Bioinformatics and Molecular Botany, Friedrich Schiller University Jena, 07743 Jena, Germany; Marine Botany, Faculty of Biology and Chemistry, University of Bremen, 28359 Bremen, Germany; Department of Fisheries and New Biomarine Industry, SINTEF Ocean AS, 7465 Trondheim, Norway; Seaweed Solutions AS, Bynesveien 50C, 7018 Trondheim, Norway; Marine Botany, Faculty of Biology and Chemistry, University of Bremen, 28359 Bremen, Germany; CIBIO, Research Centre in Biodiversity and Genetic Resources – InBIO Associate Laboratory, Campus of Vairão, University of Porto, Vairão, Portugal; BIOPOLIS Program in Genomics, Biodiversity and Land Planning, CIBIO, Campus of Vairão, Vairão, Portugal

**Keywords:** acclimation, biogeography, climate change, local adaptation, macroalgae, marine ecology, metabolites, molecular biology, omics, physiology, seaweed, warming

## Abstract

**Background:**

The sugar kelp *Saccharina latissima* is a Laminariales species widely distributed in the Northern Hemisphere. Its physiology and ecology have been studied since the 1960s, given its ecological relevance on western temperate coasts. However, research interest has been rising recently, driven mainly by reports of negative impacts of anthropogenically induced environmental change and by the increased commercial interest in cultivating the species, with several industrial applications for the resulting biomass.

**Scope:**

We used a variety of sources published between 2009 to May 2023 (but including some earlier literature where required), to provide a comprehensive review of the ecology, physiology, biochemical and molecular biology of *S. latissima*. In so doing we aimed to better understand the species’ response to stressors in natural communities, but also inform the sustainable cultivation of the species.

**Conclusion:**

Due to its wide distribution, *S. latissima* has developed a variety of physiological and biochemical mechanisms to adjust to environmental changes, including adjustments in photosynthetic parameters, modulation of osmolytes and antioxidants, reprogramming of gene expression and epigenetic modifications, among others summarized in this review. This is particularly important because massive changes in the abundance and distribution of *S. latissima* have already been observed. Namely, presence and abundance of *S. latissima* has significantly decreased at the rear edges on both sides of the Atlantic, and increased in abundance at the polar regions. These changes were mainly caused by climate change and will therefore be increasingly evident in the future. Recent developments in genomics, transcriptomics and epigenomics have clarified the existence of genetic differentiation along its distributional range with implications in the fitness at some locations. The complex biotic and abiotic interactions unraveled here demonstrated the cascading effects the disappearance of a kelp forest can have in a marine ecosystem. We show how *S. latissima* is an excellent model to study acclimation and adaptation to environmental variability and how to predict future distribution and persistence under climate change.

## INTRODUCTION

Kelps, in the strict sense including only representatives of the order Laminariales, are brown macroalgae (Phaeophyceae) growing on shallow rocky shores along the Atlantic, Pacific and Indian Oceans (Wernberg *et al.*, 2019). In the Northern Hemisphere, kelps are represented mainly by the genera *Alaria*, *Laminaria* and *Saccharina* (Bolton, 2010; Wernberg *et al.*, 2019). Kelps have received considerable attention, given their ecological roles, the several ecosystem services they provide and the several commercial applications of their extracts (e.g. Bartsch *et al.*, 2008; Smale *et al.*, 2013). Recently, threats to kelp persistence around the globe have been reviewed, and the need for conservation measures has been reiterated (e.g. Filbee-Dexter *et al.*, 2019; Smale, 2020; Filbee-Dexter and Wernberg, 2018).

**Fig. 1.**
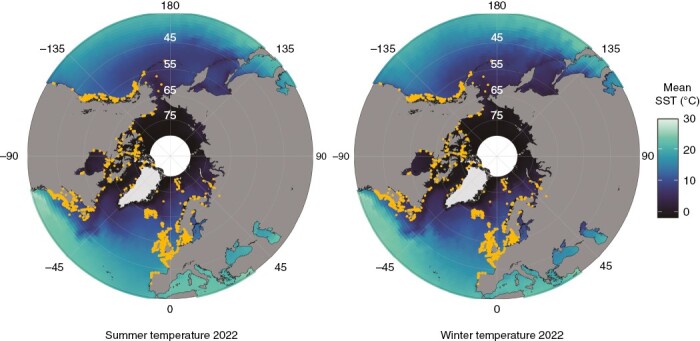
The worldwide distribution of *Saccharina latissima*. Occurrence data of *S. latissima* (orange dots) were collected from databases [Global Biodiversity Information Facility (www.gbif.org) and the Ocean Biogeographic Information System (http://iobis.org)]. Occurrence data cover the time frame between 1903 and 2020. Note that the points size is increased to allow visualization at this large scale and does not display the real areal extent Sea surface temperature data (colour gradient) from 2022 [left panel, summer temperature (21 March to 21 September 2022); and right panel, winter temperature (1 January to 21 March 2022 and 21 September to 31 December 2022)] were downloaded from the NOAA database (https://coastwatch.pfeg.noaa.gov/erddap/). The maps integrate the monthly temperature mean with latitude and longitude averaged as integers. There are white areas around the North Pole, where the projection makes data interpolation impossible. Maps were created with the R package ggOceanMaps (Vihtakari, 2022).

Among kelps, *Saccharina latissima* (Linnaeus) C.E. Lane, C. Mayes, Druehl & G.W. Saunders (Lane *et al.*, 2006) is one of the most well studied, especially in more recent years. *Saccharina latissima* is a boreal–temperate kelp widely distributed across the Northern Hemisphere, from polar to temperate regions (Fig. 1). Given its wide distribution range covering highly distinct climatic regions, this species is a brilliant model to understand environmental and adaptation. Moreover, given that it contains several valuable metabolites for the industry, research on its biochemical composition is well developed and provides an understanding on how mechanisms work at the metabolome level for kelps. Recently, ‘-omics’ tools have been developed and applied in *S. latissima* as researchers try to understand the genetic diversity that underlies the adaptation of *S. latissima* to different temperature, salinity and light regimes. This, in the context of climate change, which is precipitating the retreat and local extinction of several kelps, results in *S. latissima* being an excellent model to understand resilience and adaptation in brown algae.

This review (part I) focuses on knowledge generated over the past ~15 years, particularly on recent developments that provide new insights into the physiology and ecology of *S. latissima*. It is divided into six main themes, with a final ‘*Conclusions*’ section highlighting the needs for future research. For a review of previous work, we refer the reader to Bartsch *et al.* (2008). The second part of the review (The sugar kelp *Saccharina latissima* II: recent advances in farming and applications) focuses on the latest applied research, farming and applications for *S. latissima (Saether *et al.* 2023)*.

### Life cycle and phenology

**Fig. 2.**
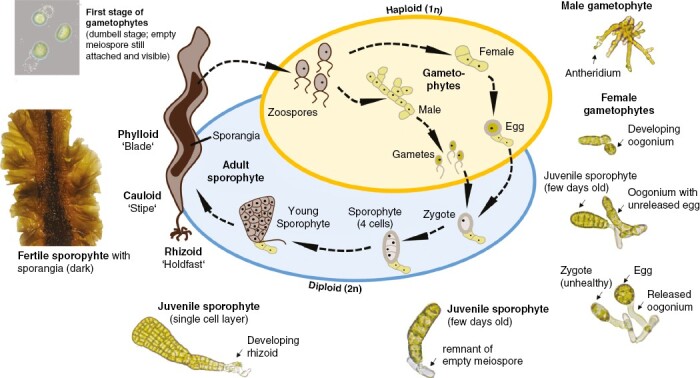
Life cycle of *Saccharina latissima*. The life cycle of *S. latissima* can be split into a diploid phase (blue) and a haploid phase (yellow). Adult sporophytes (2*n*) release zoospores, which grow into either female or male gametophytes (1*n*). Female gametophytes release eggs (1*n*); male gametophytes release gametes (1*n*). The egg and gamete fuse to form a zygote (2*n*), which grows into a sporophyte (2*n*). Sporophyte photograph: S. Forbord. Microscopic photographs and description (I. Bartsch) are included to provide additional information about the variety and diversity of gametophytes.

**Fig. 3.**
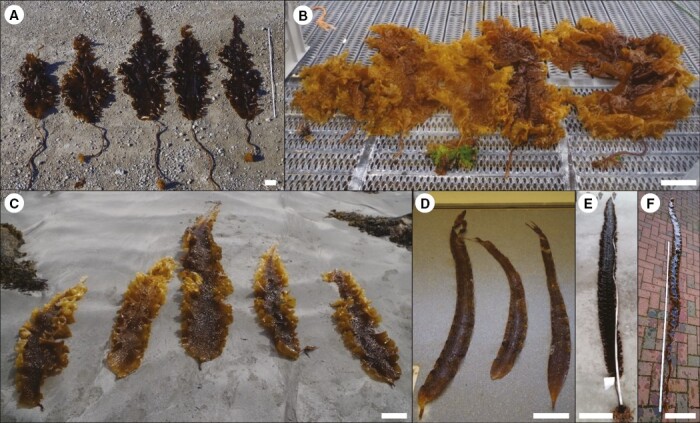
Morphological variability of European *Saccharina latissima* sporophytes. The white bars represent 20 cm. (A) Ny-Ålesund, Spitsbergen; collected from the Old Pier, 10 m depth, moderate exposure (photograph: N. Diehl). (B) Ansnas, Norway; collected in a small bay, 1–2 m depth, protected (photograph: N. Diehl). (C) Runde, Norway; collected from rocks surrounded by sand, 1–2 m depth, moderate exposure (photograph: N. Diehl). (D) Runde, Norway; collected in a *Laminaria digitata* forest, 1–3 m depth, exposed (photograph: N. Diehl). (E) Locmariaquer, France; collected from rocky shores, high tidal range, 3–5 m depth, moderate exposure (photograph: L. Fouqueau). (F) Helgoland, Germany; collected from rocky shores, 5 m depth, exposed (photograph: A. Wagner). Figure modified from Diehl *et al.* (2023).

*Saccharina latissima*, like all Laminariales, is characterized by a haplo-diplontic (haploid–diploid) heteromorphic life cycle (Fig. 2; Schiel and Foster, 2006; Bartsch *et al.*, 2008). Sessile macroscopic sporophytes (2*n*) of *S. latissima* usually grow up to 4 m (White and Marshall, 2007) and vary greatly in their morphological appearance (Fig. 3; Diehl *et al.*, 2023). Bigger specimens can be found in Arctic regions (~7 m and larger; T. Vonnahme & S. Niedzwiedz, pers. comm., June 2023). The species grows typically on rocky shores in the upper subtidal zone to depths of 15–30 m, attached to hard rock using a branched claw-like holdfast (Pehlke and Bartsch, 2008; Bekkby and Moy, 2011; Bischof *et al.*, 2019). It has also been reported growing on fine sediment attached to sparse gravel, pebbles (Bluhm *et al.*, 2022; Filbee-Dexter *et al.*, 2022b) and on tubeworms (Bracken, 2018). The sporophyte of *S. latissima* changes greatly in morphology depending on exposure and environmental factors (Fig. 3; Lüning, 1990; Van den Hoek *et al.*, 1995). In general, the phylloid is elongate, undivided and without a midrib, but may have bullations (wrinkled surface) and wavy rims (White and Marshall, 2007). Under moderate wave exposure, *S. latissima* develops narrow fronds and solid cauloids (Lüning, 1990; Van den Hoek *et al.*, 1995). In addition, sporophytes tend to develop longer and heavier stipes at greater depths to enhance light capture (Ronowicz *et al.*, 2022). This morphological plasticity has led to misidentification and taxonomic confusion. For example, *Saccharina angustissima* has only recently been elevated to species level, being until then considered a morphotype of *S. latissima* (Augyte *et al.*, 2018), whereas both *Saccharina longicruris* and *Saccharina groenlandica* were synonymized with *S. latissima* (McDevit and Saunders, 2010; Longtin and Saunders, 2015). The adult sporophyte exhibits basal meristematic growth. Sporophytes normally have a lifespan of 3 years, reaching their maximum size in the second growing season (Lee, 1989). However, specimens in the intertidal zone are annuals (Lee, 1989).

When mature, sporangia accumulate into easily recognizable sori on sporophytes of *S. latissima* and produce microscopic spores (*n*) (Fig. 2; Forbord *et al.*, 2012). As free-living stages, spores and gametes are the phases that allow for dispersal, albeit usually limited to a few metres in kelps. Therefore, spores tend to settle near parent sporophytes (Schiel and Foster, 2006). Sex is expressed at the haploid stage, and gametes and gametophytes present sexually dimorphic traits. Female gametophyte cells and nuclei are larger and rounder, whereas male gametophytes cells are smaller and tend to form filaments with more cells (Lüning and Neushul, 1978; Goecke *et al.*, 2022) which allows for identification and separation of sexes in the laboratory.

After the seminal work in the 1970s and 1980s by Lüning in Europe and by Lee and Brinkhuis in North America (e.g. Lüning, 1980; Bolton and Lüning, 1982; Lee and Brinkhuis, 1988), research targeting the sexual reproductive stages of *S. latissima* stalled. Recently, the research interest has risen again, driven by the need to manipulate the sexual life cycle in aquaculture. Hence, recent advances have enabled researchers to control the reproductive period artificially in the laboratory at several stages, allowing for scientific experimentation and improving the economic sustainability of seaweed aquaculture (Charrier *et al.*, 2017). Also, methodological advances have allowed better examination of the development of embryos to study cellular interactions in the embryo (Clerc *et al.*, 2022), quantify DNA content in different cell types (Goecke *et al.*, 2022) and improve protocols for studying embryogenesis (Theodorou *et al.*, 2021).

At the spore stage, sporogenesis (production of spores) in the wild typically peaks during winter, being negligible in summer; however, the extent of the sorus formation period is dependent on the geographical region (Bartsch *et al.*, 2008; Andersen *et al.*, 2011; Boderskov *et al.*, 2021). In the laboratory, sporogenesis is commonly induced by applying short-day light treatments, mimicking the light conditions of autumn/winter, and by removing the basal blade of the meristem, to remove inhibitors, ensuring year-round spore availability for farmers and researchers (Forbord *et al.*, 2012). In turn, a recent study reported higher and faster induction of sporulation in tissues under complete darkness than in short-day treatments (Boderskov *et al.*, 2021).

At the gametophyte stage, gametogenesis (maturation) can be induced or prevented by manipulating both biotic and abiotic conditions (see below on the next paragraph). When gametogenesis is prevented, gametophytes remain vegetative and continue to grow, remaining viable for several years [≥1 year reported in *S. latissima* (Ebbing *et al.*, 2021*b*); ≤30 years in several *Laminaria* sp. (Druehl *et al.*, 2005; Martins *et al.*, 2019)], also referred to as delayed gametophytes. Cultures of delayed gametophytes can function as genetic diversity reservoirs if conserved by cryopreservation, which has been applied successfully to the gametophytes of *S. latissima* (Visch *et al.*, 2019). In parallel, vegetative growth of gametophyte cultures can be boosted to produce enough biomass for cultivation facilities. In the wild, delayed gametophytes might represent a marine resource analogous of terrestrial seed banks, preserving the algae in a resting stage during harsh environmental conditions and allowing for a rapid recovery once the conditions improve (Schiel and Foster, 2006). However, the high levels of gene expression reported in vegetative gametophytes indicate that these gametophytes are metabolically active and not resting stages where growth is stopped, calling for more research on the topic (Monteiro *et al.*, 2019*a*). Recent methodological advances, such as the use of flow cytometry to isolate gametophytes of *S. latissima*, will allow for a more cost-effective gametophyte control at a larger scale (Augyte *et al.*, 2020). For more information on aquacultural approaches, see Review II (Saether *et al.*, 2023).

The maturation of female gametophytes depends on the interaction of temperature, light quality and intensity, nutrients and biotic factors. Blue light is required for female gametophytes to mature, and as temperature rises, more blue light is required until an inhibitory species-specific threshold: 20 °C in *S. latissima* (Lüning and Dring, 1972; Lee and Brinkhuis, 1988). Therefore, in laboratory conditions, if only exposed to red light, gametophytes will tend to grow vegetatively, because growth is unaffected by light quality (Lüning and Dring, 1975). Recently, a study revealed that light quality was significant only at lower intensities; at higher intensities, both red and blue light induced maturation (Ebbing *et al.*, 2021*b*).

Concerning nutrients, it has been shown that iron is necessary for oogenesis in kelps; hence, iron is typically excluded from nutrient solutions given to stock culture meant to grow vegetatively (Motomura and Sakai, 1981; Lewis *et al.*, 2013). Also, nutrient enrichment favours gametophyte growth; however, caution must be taken with the proliferation of diatoms, the growth of which is inhibited by addition of germanium dioxide (Kerrison *et al.*, 2016; Nielsen *et al.*, 2016*a*).

Concerning biotic factors, concentrations above an optimal initial density of gametophytes inhibit fertilization, regardless of temperature and light intensities (Ebbing *et al.*, 2020). The authors ruled out reduced nutrients or light intensity as the cause of inhibition of fertilization at high concentrations; hence, the underlying mechanism remains unknown. Another relevant biotic factor is the sex ratio of cultures, with a higher proportion of female gametophytes decreasing the reproductive yield, most relevant at high culture densities (Ebbing *et al.*, 2021*a*).

Concerning phenology, in the wild, the maturation process of *S. latissima* typically peaks in winter, with sporophytes growing at the highest rate over spring, after which they often senesce over summer owing to high temperatures. However, in some sites, the species produces sori throughout the year (Boderskov *et al.*, 2021). Although reproduction can occur over several months, reproductive success and sporophyte growth depend on the month when sporogenesis occurs. In Denmark (temperate Atlantic), the percentage of fertile sporophytes (with visible sorus formation) varies markedly over the year, peaking in November and December and reaching null values in July and September. The number of viable spores released also varies monthly, decreasing steadily from a maximum in November to February, with a surge in March and April (Boderskov *et al.*, 2021). Meiospores of *S. latissima* (from Alaska, USA; Arctic Pacific) released in July resulted in larger gametophytes but smaller sporophytes when compared with spores released in August (Raymond and Stekoll, 2021), whereas for spores originated from *S. latissima* collected in April (from Ireland, temperate Atlantic), growth rates of gametophytes were five to ten times higher than from spores originated in February (Nielsen *et al.*, 2016*a*).

Concerning sporophyte growth, seasonal variation in growth rates is notable along the coast of Norway, with sporophytes from northern Norway reaching their maximum frond length and biomass ~2 months earlier than sporophytes occurring in the south of the country (Forbord *et al.*, 2020).

The fact that recent studies (Ebbing *et al.*, 2020; Boderskov *et al.*, 2021) sometimes contradict previous findings and/or show a more complex control of life-cycle transitions highlights the need for further research on this topic, testing for more single and interacting drivers and accounting for possible site-specific responses.

## ADVANCES IN ‘OMICS’

### Genomics

The decrease in sequencing costs has led to an increase in genomic resources for non-model species, such as brown algae, that have been severely understudied until recently. Nuclear genomes are now available for some Phaeophyta species [e.g. *Ectocarpus* sp. (Cock *et al.*, 2010), *Saccharina japonica* (Ye *et al.*, 2015; Liu *et al.*, 2019), *Undaria pinnatifida* (Shan *et al.*, 2020; Graf *et al.*, 2021)], and plastid and mitochondrial genomes are also mounting (e.g. Oudot-Le Secq *et al.*, 2006; Chen *et al.*, 2019; Rana *et al.*, 2021). For *S. latissima*, a mitochondrial genome is available (Wang *et al.*, 2016) but not a nuclear genome, although efforts are underway (M. Cock, pers. comm.; https://phaeoexplorer.sb-roscoff.fr/home/). Based on genetic data, a taxonomic re-organization was proposed in 2006 that reassigned the previously *Laminaria saccharina* to *Saccharina latissima*, the currently accepted species name (Lane *et al.*, 2006). Since then, other species have been synonymized with *S. latissima* (Neiva *et al.*, 2018), highlighting the need for more extensive sampling across described and possible sites where *S. latissima* occurs to assess the intraspecific diversity better. The availability of validated DNA barcodes for the species [mitochondrial cytochrome *c* oxidase I (*COI*) and ribulose-1,5-bisphosphate carboxylase/oxygenase large subunit (rbcL) (Ratnasingham and Hebert, 2007)] is important to confirm the identity of *S. latissima* samples. Moreover, it allows for the species to be detected in environmental DNA surveys, which allow for identification and quantification of several species from a unique sample using metabarcoding techniques (Deiner *et al.*, 2017). Population structure, connectivity and genetic diversity in *S. latissima* have been studied using microsatellites (e.g. Guzinski *et al.,* 2016; Luttikhuizen *et al.*, 2018; Mooney *et al.*, 2018), *COI* (Neiva *et al.*, 2018) and, more recently, double digest restriction site-associated DNA sequencing, and the results are discussed in the section entitled ‘*Biogeographical patterns*’. Using a genomic selection approach, breeding values of the gametophytes of *S. latissima* were estimated and correlated with phenotypic traits of sporophytes, especially wet and dry weight per metre; however, low genetic correlations among different years are concerning and need to be explored further (Huang *et al.*, 2023). These approaches inform current attempts to establish breeding programmes and, in the future, to domesticate *S. latissima* (Yarish *et al.*, 2017; Umanzor *et al.*, 2021).

### Transcriptomics

Responses of organisms to stress are often measured by physiological parameters, such as survival, reproductive success or growth, which are extremely relevant because they underlie the success of species. However, the underlying molecular mechanism often remains unknown even when significant physiological responses are found after exposure to a stressor (Bischof *et al.*, 2019). Transcriptomics approaches focus on the expression of mRNA following a stimulus. Given the nature of mRNA, this approach measures a transient response that can be encoded at the DNA level or via epigenetic mechanisms (Stark *et al.*, 2019). The application of this approach to non-model organisms has been rising in recent years, and methods have improved considerably in a short period. Nonetheless, the use of transcriptomics in brown algae is lagging and has been applied to only a few species [e.g. *Laminaria digitata* (Liesner *et al.*, 2022); *Undaria pinnatifida* (Graf *et al.*, 2022); and mostly on the brown algal model *Ectocarpus* (e.g. Ahmed *et al.*, 2014; Mignerot *et al.*, 2019) and the commercially important *Saccharina japonica* (e.g. Liu *et al.*, 2014; Zhang *et al.*, 2021)]. Although access to transcriptomic data in brown algae has been made easier by advances in (higher) model plants, namely *Arabidopsis thaliana* (e.g. Zhang *et al.*, 2017), the evolutionary distance between Phaeophyceae and plants and other algae creates challenges. There is still a very low annotation rate of expressed genes in brown algae because few functional studies have been conducted in this group, given that approaches such as reverse genetics are not available (Kroth, 2013; Bringloe *et al.*, 2020). However, promising advances have been made recently, and the use of CRISPR/Cas9 technology might enable a better understanding of the function of each gene in the metabolism of this group (Badis *et al.*, 2021).

Gene-expression patterns in *S. latissima* were investigated initially using microarrays (Heinrich *et al.*, 2012*a*, *b*; Heinrich *et al.*, 2015, 2016), but more recently, RNA-sequencing has been applied (Monteiro *et al.*, 2019*a*, *b*; Pearson *et al.*, 2019; Li *et al.,* 2020*a*; Li *et al.*, 2020*b*), and reference genes for real-time quantitative PCR have been developed (Xing *et al.*, 2021). Transcriptomic studies in *S. latissima* have revealed an intricate metabolism-wide programming of gene expression in the species in response to environmental drivers, discussed in the section ‘*Responses to environmental drivers*’.

### Epigenomics

Epigenomics have been shown to play a crucial role in defining a phenotype (Moore *et al.*, 2013; Anastasiadi *et al.*, 2021). Given its sessile lifestyle and often low dispersal distances, *S. latissima* is likely to rely on epigenetic mechanisms and epigenetic variation. Epigenetic mechanisms play an essential role in the adaptation of a population, and in the coping mechanisms of an individual in reaction to local conditions, ecotype differentiation (eco-phenotype) or rapid changes in local conditions (= local).

The known, non-exclusive epigenetic mechanisms encompass non-coding RNA, histone modification and DNA methylation (Boquete *et al.*, 2021). They have been shown to play a role in establishment, maintenance and control of gene expression without changes to the DNA sequence (Anastasiadi *et al.*, 2021); hence, they play a key role in the eco-evolutionary dynamics of a species (Calosi *et al.*, 2016; Anastasiadi *et al.*, 2021). Research on epigenetic modulation and variation thereof is well established in plant biology (Richards *et al.*, 2017). However, in kelp, the study of epigenetics has recently gained momentum, with only a handful of studies to date (Phaeophyceae; Cock *et al.*, 2010; Liu *et al.*, 2019; Fan *et al.*, 2020*a*; Teng *et al.*, 2021; Scheschonk *et al.*, 2022).

Regarding epigenetic mechanisms in the genus *Saccharina*, only DNA cytosine methylation has been investigated so far (Liu *et al.*, 2019; Fan *et al.*, 2020*a*; Teng *et al.*, 2021; Scheschonk *et al.*, 2022). ‘DNA methylation’ in plants and algae describes the methylation of a cytosine in the DNA (5ʹ-methylcytosine). DNA cytosine methylation can occur within and outside genes in the sequence context of CG, CHG or CHH (with ‘H’ being any base except G; Bewick *et al.*, 2017). Genes are typically methylated in the CG context in animals (Schmitz *et al.*, 2019), and methylation of the CG context in gene bodies of nuclear DNA is between 2 and 86 % across Viridiplantae (Bewick *et al.*, 2017). Methylations in the CG, CHG and CHH contexts were found to act in silencing transposable elements in and outside of genes (Zhou *et al.*, 2020) or to act in regulation of transcript expression (Dubin *et al.*, 2015; L. Zhang *et al.*, 2018*a*; Boquete *et al.*, 2021). With this, they are important to consider as aspects of and adaptation processes. Moreover, it has been proposed in plants that CG methylation regulates the inheritance of other types of epigenetic information (Mathieu *et al.*, 2007).

Within brown algae, there seem to be group-specific occurrences regarding the types of epigenetic mechanisms. Histone modification has been observed in *Ectocarpus siliculosus* (Cock *et al.*, 2010; Bourdareau *et al.*, 2021), whereas DNA cytosine methylation was found to be negligible, which led to the assumption that DNA methylation is negligible in brown algae (Cock *et al.*, 2010). However, in the kelps *S. latissima* and *S. japonica*, it has recently been established that methylation plays a significant role in gene expression, for both the nuclear genome and the chloroplast genome (e.g. Fan *et al.*, 2020a, b; Yang *et al.*, 2021; Scheschonk *et al.*, 2022). Hence, it is likely that the totality of epigenetic modifications of importance in *S. latissima* can be assessed only by testing for the respective mechanism in the species, or possibly the congener species (*S. japonica*), but cannot be implied per se by findings from other genera within the group of Phaeophyceae. The studies focusing on *Saccharina spp.* investigated the impact of cytosine methylation on both life-cycle stages at the transcriptomic level (*S. japonica*; Liu *et al.*, 2019; Fan *et al.*, 2020; Teng *et al.*, 2021) and differences in cytosine methylation attributable to cultivation and latitudinal location (possibly heritable traits) observable at the sporophyte stage (*S. latissima*; Scheschonk *et al.*, 2022; L. Scheschonk, unpubl. res.). Cytosine methylation was shown to influence gene expression in both life-cycle stages (predominantly, the non-heritable methylation variant CHH; ~56 %; Yang *et al.*, 2021), with higher methylations found in the gametophyte stage for both nuclear and chloroplast genome (Fan *et al.*, 2020*b*; Teng *et al.*, 2021). In both life-cycle stages and genomes (nuclear and chloroplast), high levels of cytosine DNA methylation led to the silencing of the respective DNA sequence, acting as an additional control mechanism in gene expression (Fan *et al.*, 2020*a*). At the population level, differences in cytosine methylation were observed between latitudes in populations regardless of cultivation status (laboratory and wild; Scheschonk *et al.*, 2022; L. Scheschonk, unpubl. res.). This implies hereditary additional control imposed via cytosine methylation. As in other sequences, regions became methylated only during the cultivation process in both origins, and DNA cytosine methylations are likely to be a mechanism of rapid adaptation, because changes in habitat (wild to cultivation) initiated epigenetic changes within a generation.

## RESPONSES TO ENVIRONMENTAL DRIVERS

### Temperature

The composition and biogeographical distribution patterns of macroalgal communities are largely determined by temperature (Lüning, 1984; Adey and Steneck, 2001; Wiencke and Bischof, 2012). Thus, climate change, particularly warming and marine heatwaves (MHWs), is a major threat to marine forests (e.g. Harley *et al.*, 2012; Smale, 2020). Hobday *et al.* (2016) defined MHWs as a temperature increase above the 90th percentile of the 30-year mean for >5 days consecutively; however, several publications mention MHWs as prolonged anomalously warm water events. As the use of the term ‘MHW’ differs among studies, in this review, we refer to the wording of the individual studies.

Much is known about the general thermal characteristics of *S. latissima*, mainly in terms of survival, reproduction, photosynthesis and growth (Bartsch *et al.*, 2008). Like other kelps, *S. latissima* is a cold-temperate organism (Araújo *et al.*, 2016). The survival threshold of sporophytes has been shown to be location specific. Sporophytes from Helgoland presented optimal growth between 10 and 15 °C (Bolton and Lüning, 1982), although they tolerated an extensive range of temperature, from 0 to 23 °C, for shorter periods, with sharply increasing mortality rates at >20 °C (Fortes and Lüning, 1980; Lüning, 1984, 1990a). Sporophytes from Nova Scotia were found to have decreasing growth rates with increasing temperatures between 11 and 21 °C, high mortality at 18 °C and no survival at 21 °C after merely 2 weeks (Simonson *et al.*, 2015*a*). On the contrary, *S. latissima* sporophytes from Brittany survive ≤25 °C for >1 week (Diehl *et al.*, 2021). Susceptibility to high temperature was shown to vary with environmental thermal history, thus between seasons and years (Niedzwiedz *et al.*, 2022). Gametophytes of *S. latissima* exhibited a broader thermal tolerance, surviving temperatures down to −1.5 °C and up to 23–25 °C (tom Dieck, 1993). Differences in temperature sensitivity were also found between laboratory cultures and field sporophytes (Heinrich *et al.*, 2016) and between male and female gametophytes (Monteiro *et al.*, 2019*a*). Consequently, generalizations about thermal limits of *S. latissima* based on limited spatial covering and without consideration of generational effects should be handled carefully.

Detrimental effects of suboptimal high temperatures on *S. latissima* often include compromised growth (e.g. Bolton and Lüning, 1982; Simonson *et al.*, 2015b), but high temperature can also lead to weakening of the tissue structure (Simonson *et al.*, 2015*b*), increasing blade erosion (Krumhansl *et al.*, 2014; Simonson *et al.*, 2015*b*), enhanced biofouling and epiphytism (Andersen *et al.*, 2013; Forbord *et al.*, 2020), complex modifications in photosynthetic mechanisms, lowered chlorophyll *a* and fucoxanthin concentrations (Andersen *et al.*, 2013), a strongly increased de-epoxidation state of the xanthophyll cycle (Nepper-Davidsen *et al.*, 2019; Diehl *et al.*, 2021) and reduced kelp carbon decomposition (Filbee-Dexter *et al.*, 2022a). Exposure to elevated, although not lethal, temperature is harmful in the long term for *S. latissima* (Andersen *et al.*, 2013; Nepper-Davidsen *et al.*, 2019). Warming in the Arctic, however, might promote kelp populations, with densities being higher in warmer areas than at comparable colder sites (Wiktor *et al.*, 2022). At the warmer sites, *S. latissima* was also found at slightly greater depths.

It is increasingly relevant to look at the impact of MHWs on seaweeds (Straub *et al.*, 2019). Strong correlations between MHW events over the last 60 years and loss of *S. latissima* forests in the East and West North Atlantic were found (Filbee-Dexter *et al.*, 2020). Nevertheless, few studies simulating MHW scenarios have been conducted on *S. latissima* (see Nepper-Davidsen *et al.*, 2019; Diehl *et al.*, 2021; Niedzwiedz *et al.*, 2022). After a simulated 3-week MHW event in Danish waters, most samples died within a few days at 24 °C, and impairing effects of high but sub-lethal temperatures (18 and 21 °C) were observed in a 2-week recovery phase (Nepper-Davidsen *et al.*, 2019). Thereby, interrelationships were demonstrated between reduced growth, reduced photosynthetic performance, carbon uptake and pigment composition. At the same temperatures (11, 18 and 21 °C), no changes in C:N and phlorotannins were detected in specimens from Nova Scotia, Canada (Simonson *et al.*, 2015*b*). The impact of local MHWs in the summer on five European *S. latissima* populations ranging from southern Brittany to Spitsbergen revealed strong physiological and biochemical divergences between the populations. Increased mortality and decreased photosynthetic performance at the higher temperature amplitude treatments were detected exclusively in the rear-edge populations from Helgoland (German Bight) and Brittany, while the Arctic population was unaffected (Diehl *et al.*, 2021). In Norway, strong differences in the physiological condition of *S. latissima* were observed, showing, e.g. decreased growth and more erosion in a hot year compared with a cooler year (Armitage *et al.*, 2017). The impact of MHWs also varies by year and season, as shown for field sporophytes from Helgoland (Niedzwiedz *et al.*, 2022). *Saccharina latissima* was more sensitive to high temperatures at the end of summer and during an extremely warm year.

High and excessively low temperatures alter physiological and biochemical properties of *S. latissima*. Overall, wild *S. latissima* from Iceland revealed a positive correlation of carbohydrates and negative correlations of proteins with the environmental temperature (Coaten *et al.*, 2023). Lower pigment concentrations were found at temperatures of <10 °C, whereas the de-epoxidation state of the xanthophyll cycle was significantly higher compared with higher temperature treatments (Olischläger *et al.*, 2017; Monteiro *et al.*, 2019*b*; Li *et al.*, 2020*a*), and higher phosphorylation rates of mitogen-activated protein kinases were measured at 2 °C than at 7 °C (Parages *et al.*, 2013). Additionally, strongly enhanced mannitol concentrations were detected in young sporophytes from Brittany after 0 °C treatment, indicating a strong anti-freezing response of the species (C Monteiro *et al.*, 2020*a*). Consequently, *S. latissima* will most probably benefit from the predicted rising temperatures in subpolar and polar regions (Filbee-Dexter *et al.*, 2019; Diehl and Bischof, 2021), because the physiological functions of *S. latissima* will be enhanced (Iñiguez *et al.*, 2016). Yet, darkness during the polar night seems to outcompete the positive effects of warming (Scheschonk *et al.*, 2019), and low water temperature is a requirement for survival (Gordillo *et al.*, 2022). Warming in winter accelerated weight loss of young sporophytes over 4 months of darkness, with ~50 % at 8 °C and 40 % at 3 °C (Gordillo *et al.*, 2022). Furthermore, dark respiration of Arctic *S. latissima* sporophytes increased with increasing temperatures (3, 7 and 11 °C) (Niedzwiedz and Bischof, 2023).

Arctic *S. latissima* gametophytes did not survive at 20 °C in the laboratory but grew at ≤15 °C, with higher growth rates between 10 and 15 °C than at 5 °C (measured as the length of both male and female gametophytes) (Park *et al.*, 2017). Another laboratory study looking at Arctic gametophytes showed that they survive at 20 °C by heat stress mechanisms that were induced extensively at the transcriptomic level at this temperature, whereas this was not the case at 4 and 12 °C (Monteiro *et al.*, 2019*a*).

Considering spore germination, a higher temperature of 9 °C increased the germination rate of spores compared with 5 °C for Arctic individuals (Zacher *et al.*, 2016). In an experiment with individuals from North America, at temperatures between 4 and 12 °C, lower temperatures negatively influenced the size of gametophytes and sporophytes and the production of eggs and young sporophytes (Raymond and Stekoll, 2021). When looking at sexual reproduction, sex-biased responses to temperature were found, with male gametophytes being more resilient to higher temperatures than females; females grew at a slower rate, and pathways related to fecundity were repressed (Monteiro *et al.*, 2019*a*). Likewise, higher temperatures increased the proportion of male gametophytes in an earlier study (Lee and Brinkhuis, 1988), but not more recently (Park *et al.*, 2017).

Recently, the impact of increasing temperatures in the Arctic, in combination with decreased salinity (Monteiro *et al.*, 2019*b*; Diehl and Bischof, 2021), increased partial pressure of CO_2_ (*p*CO_2_) (Olischläger *et al.*, 2014, 2017; Iñiguez *et al.*, 2016), ultraviolet (UV) radiation stress (Parages *et al.*, 2013), increased sedimentation (Zacher *et al.*, 2016) or increased nutrient conditions (Diehl and Bischof, 2021), were investigated. All these studies showed that growth, photosynthetic performance, biochemical composition and the transcriptomics of *S. latissima* were strongly affected by temperature. The species would benefit from higher temperatures in Arctic regions, whereas the impact of the other drivers was less pronounced or there was no impact at all. On the contrary, the early stages of *S. latissima* appear vulnerable to strong warming and interaction with other factors in the Arctic. Overall, strong interactions between light and temperature were also detected in different microstages, highlighting the impairing effect of UV-B radiation (Müller *et al.*, 2008, 2012). Increased production of superoxide anion radicals was measured in gametophytes in increasing temperatures between 2 and 18 °C and slightly under UV radiation (Müller *et al.*, 2012). Temperatures ≤21 °C combined with hyposalinity diminished the spore settlement of *S. latissima* from Alaska (Lind and Konar, 2017). Although higher temperatures generally lead to higher germination rates of Arctic *S. latissima* spores, temperature and grazing had an interactive effect (Zacher *et al.*, 2016). At 5 °C, the germination rate was higher when grazers were present, whereas at 9 °C, the reverse happened. The same pattern holds for the density of juvenile sporophytes. The species-specific interactive effects revealed a differential response between co-occurring kelps in the Arctic.

Large ecosystem shifts from *S. latissima* canopies or dominance to turfs or barrens have been reported. Generally, the loss of *S. latissima* populations has been attributed to warming to a certain extent. In Norway, *S. latissima* communities were observed to be replaced by ephemeral, filamentous turf algae (Moy and Christie, 2012; Christie, Andersen *et al.*, 2019). This ecosystem shift was proposed to have been driven mainly by extraordinarily high temperatures over summer, in combination with eutrophication (Moy and Christie, 2012). Loss of *S. latissima* beds and shifts to turf-dominated ecosystems were also observed in Nova Scotia, Canada, caused by increased temperature and diverse unbalanced multitrophic interactions (Filbee-Dexter *et al.*, 2016).

The impacts of interactions between MHWs and biota on kelp forests appear to be extremely dynamic and complex. Although the severe declines of *S. latissima* in the eastern and western North Atlantic were attributable primarily to large increases in the frequency and cumulative intensity of MHWs, excluding alternative effects, such as turbidity or biological factors (Filbee-Dexter *et al.*, 2020), kelp forest mortality and recovery in other regions were found also to be controlled in a top-down manner (Christie *et al.*, 2019*b*; Norderhaug *et al.*, 2021). Thus, multifactorial experimental set-ups are of major importance in identifying the complexity of reactions to climate change and local anthropogenic stressors (Strain *et al.*, 2014). Overall, much research has been done on Arctic and Norwegian populations of *S. latissima*. In contrast, the knowledge about the potential of southern populations is scarce and should receive particular attention in future studies.

### Hydro-optics

As photosynthetic organisms, seaweeds are dependent on light availability to survive. Irradiance effects on *S. latissima* have already been well studied for decades and have been summarized by Bartsch *et al.* (2008). Both extremely high and low photosynthetic active/available radiation (PAR) and mainly UV radiation (UVR) cause modifications in multiple biochemical and physiological processes in *S. latissima*, with early life stages and adult sporophytes showing differences in susceptibility.

More recent studies have demonstrated that reduced irradiance negatively affects the growth performance of sporophytes *in situ* (Spurkland and Iken, 2011; Forbord *et al.*, 2020) without diminishing the photosynthetic performance (Spurkland and Iken, 2011) but still promoting biofouling (Forbord *et al.*, 2020). The maximum modelled distribution depth of *S. latissima* in Arctic fjords followed the extent of the meltwater plume, being shallower close to the glaciers and deeper in outer fjord regions (Niedzwiedz and Bischof, 2023). Pronounced variability was found in different parts of the phylloid regarding the long-term storage of the carbohydrate laminarin in Arctic field sporophytes between October and early February (Scheschonk *et al.*, 2019). Also, other biochemical components, such as mannitol or nitrogen, declined greatly during the dark season. Interestingly, darkness appeared to be optimal for artificial sporogenesis of Danish *S. latissima* compared with other light levels (20–120 μmol photons m^−2^ s^−1^) (Boderskov *et al.*, 2021).

A few studies suggest that other response variables, beyond the main physiological and biochemical parameters, are involved in to variations in light in *S. latissima*. Enhanced release of organic iodine and reduced release of reactive organic bromine and chlorine were found after PAR (23 µmol photons m^−2^ s^−1^) + UVR exposure (Laturnus *et al.*, 2010). The impacts of PAR (~10 µmol photons m^−2^ s^−1^) and UVR were also investigated in chloroplasts of vegetative (non-soral) and fertile (soral) tissue of *S. latissima* (Holzinger *et al.*, 2011). The fertile tissue cells were not affected by PAR + UVR, whereas negative effects were found in vegetative parts. For instance, decreased optimal quantum yields of photosystem II *F*_v_/*F*_m_ were measured under UVR treatment, and the chloroplast structure was altered, i.e. including more physodes. Another study revealed that the oxygen consumption rate of *S. latissima* was significantly higher in high light conditions (300 µmol photons m^−2^ s^−1^) compared with low light conditions (3 µmol photons m^−2^ s^−1^) (McDowell *et al.*, 2015).

Sedimentation and epibiosis have a strong impact on light availability. *Saccharina latissima* can withstand short-term sediment cover (Roleda and Dethleff, 2011; Picard *et al.*, 2022), whereas longer burial negatively affects its vitality and morphology (Roleda and Dethleff, 2011). Furthermore, it was shown that sediment from melting ice weakened the recruitment of *S. latissima* (Zacher *et al.*, 2016). Overgrowth with epibionts, with consequent shading, can reduce growth and survival of the species (Andersen *et al.*, 2018).

Polar night imposes very special conditions for Arctic *S. latissima*, especially when combined with future increases in winter temperatures. Treatments of light/dark or darkness alone seem to have a greater effect on *S. latissima* than the various temperatures applied (0, 4 or 8 °C) (Scheschonk *et al.*, 2019). The lower laminarin content at elevated temperatures (8 °C) suggests that prolonged darkness might be a problem for *S. latissima* under future temperature trends.

In a comparable study on *S. latissima* sporophytes, low temperatures (2 °C) and PAR (10 µmol photons m^−2^ s^−1^) + UVR treatments activated the rapid phosphorylation of mitogen-activated protein kinases, while UVR generally impaired the photosynthetic performance (Parages *et al.*, 2013). A study in juvenile Arctic sporophytes revealed that *F*_v_/*F*_m_ remained unchanged in low PAR treatments (~24 µmol photons m^−2^ s^−1^), even with the addition of UVR, and that it decreased under high light stress (~110 µmol photons m^−2^ s^−1^), especially when combined with UVR (Heinrich *et al.*, 2012*b*; Heinrich *et al.*, 2015). Remarkably, the photosynthetic performance exhibited particularly severe reduction at high PAR + high temperatures (17 vs. 2 and 7 °C) (Heinrich *et al.*, 2012*b*), whereas when UVR was included in a comparable set-up, the strongest inhibition occurred in the high PAR + UVR treatment at 2 °C, compared with 7 and 12 °C (Heinrich *et al.*, 2015). Thus, high temperatures appear to mitigate the impairing effects of UVR on *S. latissima* sporophytes. However, these observations were more pronounced in laboratory cultures than in field sporophytes (Heinrich *et al.*, 2015).

Investigation of the effects of irradiance (<10 and 30–50 μmol photons m^−2^ s^−1^), temperature (4, 8 or 12 °C) and season on gametophyte growth and reproduction of *S. latissima* revealed that the gametophyte length, sporophyte length, fraction of female gametophytes with eggs, and fraction of female gametophytes with sporophytes were all altered mainly by temperature and season (Raymond and Stekoll, 2021). Irradiance significantly affected all response parameters except for gametophyte length; however, interactions were found only for sporophyte length (irradiance × temperature).

In the last decade, transcriptomic responses of *S. latissima* to different light conditions have been investigated (Heinrich *et al.*, 2012*b*; Heinrich *et al.*, 2015, 2016; Li *et al.*, 2020*b*; Xing *et al.*, 2021). On the time scale of 24 h exposure, the combination of high temperature and high PAR induced more transcriptomic regulation than low temperature and low PAR. High PAR and high temperature widely downregulated genes involved in photosynthesis, including photosystem I/II components, thylakoid protein and light harvest complex proteins with strong folds (≤60-fold). Genes encoding reactive oxygen species (ROS) scavenging enzymes, oxygen heat shock proteins and proteins involved in proteolysis were upregulated under high PAR and high temperature conditions. In contrast, the combination of high PAR and low temperature generally upregulated genes encoding photosynthesis, ROS scavengers and heat shock proteins, whereas downregulated genes encoded proteolysis-related protein. Exposure to UVR for 24 h also induced a wide regulation of gene expression, mainly including photosynthetic components, DNA repair, vitamin B_6_ biosynthesis and ROS scavengers, which supported that UVR negatively affected photosynthesis and damaged DNA (Heinrich *et al.*, 2012*b*). Long-term (14 days) exposure to PAR, UVR and temperature combinations resulted in large transcriptomic reprogramming, which did not cause physiological adjustments. The combination of high PAR and UVA caused more gene regulation than the single exposure to high PAR or UVR and mainly upregulated genes encoding photosynthetic components, pigment metabolism, glycine, serine and threonine metabolism and ROS scavenging enzymes. The transcriptomic responses of *S. latissima* to 14 days of darkness at two temperatures revealed that darkness induced more regulated genes than increased temperature (Li *et al.*, 2020*b*). Darkness downregulated genes encoding enzymes involved in glycolysis and metabolite biosynthesis. Some energy-consuming processes, e.g. photosynthetic components and biosynthesis of transporters were also repressed. On the contrary, genes coding for the catabolism of lipid and laminarin, the glyoxylate cycle and signalling were upregulated in darkness, pointing out the possible energy source of *S. latissima* during the polar night.

### Salinity

Coastal salinity frequently varies with tidal ranges, precipitation, freshwater plumes from rivers or terrestrial run-offs (Lüning, 1990), increasing with climate change (Holt *et al.*, 2010; Masson-Delmotte *et al.*, 2021). Variation in salinity is particularly relevant for the physiology of *S. latissima* in Arctic fjord systems owing to enhanced sea ice and glacier melting (Hanelt *et al.*, 2001; Svendsen *et al.*, 2002; Sundfjord *et al.*, 2017). Fluctuations in salinity lead to osmotic stress, with consequences at the physiological and biochemical level, which is, overall, well studied for seaweeds (see Karsten, 2012 and references therein) but not for *S. latissima*. Although *Laminaria sensu lato* is considered a rather stenohaline genus (Bartsch *et al.*, 2008), *S. latissima* is known physiologically to tolerate broad ranges of salinities between absolute salinities (S_A_) 5 and 60 (Karsten, 2007), although young sporophytes were shown to have a tolerance of down to S_A_ 11 in laboratory conditions (Karsten, 2007; Peteiro and Sánchez, 2012), which allows the species to inhabit brackish waters (Nielsen *et al.*, 2016*c*; Mortensen, 2017). Nevertheless, hyposalinity results in decreased growth (e.g. Spurkland and Iken, 2011; Marinho *et al.*, 2015; Bruhn *et al.*, 2016; Forbord *et al.*, 2020), diminished photosynthetic performance (e.g. Karsten, 2007; Spurkland and Iken, 2011; Peteiro and Sánchez, 2012) and loss of pigmentation (Karsten, 2007; Peteiro and Sánchez, 2012). Furthermore, decreased carbon dioxide exchange rates were detected at low salinities (Mortensen, 2017). Generally, salinity has a strong effect on the biochemical composition of *S. latissima*. For instance, the content of sulfated fucose-rich polysaccharides, measured with fucoidan, generally increased at absolute salinities (S_A_ 15–25) in the Baltic Sea; however, the pattern did not hold for all locations (Bruhn *et al.*, 2017). Samples of *S. latissima* from an Atlantic population hold higher contents of fucose-containing sulfated polysaccharides than a Baltic population, which experiences lower salinity variation than the former population (Ehrig and Alban, 2015). Along the salinity gradient of the Baltic Sea’s, effects of salinity were observed in carbohydrates, proteins, pigments and nitrogen contents (Nielsen *et al.*, 2016*a*). However, it should be noted that these observations were not necessarily consistent between different populations or experimental frameworks (Manns *et al.*, 2017; Diehl *et al.*, 2023).

Little is known about the interaction between salinity and other factors in *S. latissima*, with only salinity × temperature having been investigated so far. Recent studies revealed that potentially, hyposalinity is highly stressful for *S. latissima* in combination with temperature variation. In the Baltic Sea, low salinity in combination with high summer temperatures decreases the productivity of *S. latissima* owing to high physiological stress in cultivated seaweed (Nielsen *et al.*, 2014). Arctic field adult sporophytes of *S. latissima*, however, were almost unaffected by an increase in temperature (from 4 to 10 °C) and hyposalinity (S_A_ 25) in mimicked field conditions (Diehl *et al.*, 2020), although slightly increased growth and photosynthetic performance (*F*_v_/*F*_m_) were detected at higher temperatures. In contrast to adult sporophytes, more pronounced effects of both parameters and some interaction of salinity and temperature are detectable in the early life stages of *S. latissima*. For instance, elevated temperatures and low salinities decreased spore settlement and gametophyte growth (Lind and Konar, 2017). The impact of temperature × salinity interaction was investigated in young sporophytes from Brittany and the Arctic by running comparable experiments on specimens from both locations (Monteiro *et al.*, 2019*b*; C. Monteiro *et al.*, 2020*a*; Li *et al.*, 2020*a*). Remarkably, similar effects were observed in young sporophytes from the two regions. Lower salinities had little negative impact on growth and *F*_v_/*F*_m_ and modified the xanthophyll-cycle pigment pool. The effects of different temperatures were more pronounced, revealing ameliorating effects of higher temperatures and diminishing effects of lower temperatures.

At the transcriptomic level, an ameliorating effect of high temperature was observed for algae from Brittany and Svalbard (Monteiro *et al.*, 2019*b*; Li *et al.*, 2020*a*). The treatments at low salinity (S_A_ 20) at 0 and 8 °C elicited more differentially expressed genes than at 15 °C and low salinity. Geographical variation also played an important role, because the combination of low salinity and low temperature was especially stressful for sporophytes from Brittany (not exposed to 0 °C in their environment of origin) than from Svalbard. In response to low salinity, metabolic pathways such as photosynthesis and carbon assimilation were downregulated, and some gene coding enzymes contributed to the xanthophyll cycle and cell wall metabolism were also down-regulated. Moreover, genes coding for heat shock proteins and enzymes involved in the synthesis of mannitol and proline were not significantly regulated during this experiment, perhaps revealing that the stress was mild or that the regulation of salt stress is more intricate than expected, involving several other pathways than those already described for other environmental drivers.

### Nutrients

The macronutrients nitrogen (N) and phosphorus (P) serve as essential elements for photosynthesis and growth, of which N is considered the main limiting resource for macroalgal productivity (Roleda and Hurd, 2019). An overview of nutrient physiology and factors affecting nutrient uptake in seaweeds is provided by Roleda and Hurd (2019). Effects of various nutrient regimes have been well investigated for Laminariales, including *S. latissima* (summarized by Bartsch *et al.*, 2008). Laminariales can accumulate nutrient reserves over winter when nutrient conditions are favourable (Bartsch *et al.*, 2008; Lubsch and Timmermans, 2019) and have an optimal environmental nitrate concentration of ~10 μm but also tolerate oligotrophic conditions (Kerrison *et al.*, 2015). Nutrient depletion has long been known to have negative impacts on the physiological status of *S. latissima*, resulting, for instance, in lower growth rate and lower photosynthetic performance (Williams and Herbert, 1989; Gerard, 1997*a*, *b*; Korb and Gerard, 2000; Roleda and Hurd, 2019). A recent study revealed that the development, density and growth in length of young sporophytes were also diminished in nutrient-poor conditions (Raymond and Stekoll, 2021). Nitrate uptake rates are linearly related to the substrate concentrations for both N-limited and N-saturated young sporophytes, indicating that *S. latissima* requires high ambient nitrate concentrations in the environment to produce rapid growth. Sporophytes with deficient internal nitrogen pools exhibited higher uptake rates of nitrate than sporophytes with higher internal nitrogen pools (Forbord *et al.*, 2021). As a result, the growth of *S. latissima* decreases significantly over the summer, although it can continue to grow for some time even in low nutrient conditions (Nielsen *et al.*, 2014; Lubsch and Timmermans, 2019; Forbord *et al.*, 2020). The ability of the species to store nutrients is also considered an advantage in direct competition for habitat with other seaweeds (Armitage *et al.*, 2017). Several physiological parameters of *S. latissima* are also limited by bioavailable P (Bruhn *et al.*, 2016). Comparing the effect of P enrichment on spores and gametophytes in February and April showed that growth was supported by elevated P levels (23–69 μm), and earlier gametophyte development appeared under P treatment in April (Nielsen *et al.*, 2016*a*). Sufficient or slightly enhanced N supply is reported to have beneficial effects on the response of *S. latissima* with respect to several environmental stressors. For instance, it was found that UV damage in *S. latissima* can be mitigated or prevented by enriched (50 µm) N supply (Davison *et al.*, 2007). Recent studies on nutrient × light interactions showed the high importance of nutrients (N + P). Specimens were not much altered overall by the different natural light intensities, but growth and intracellular N were positively affected by elevated nutrient conditions (Boderskov *et al.*, 2016; Jevne *et al.*, 2020). The contents of total carbon (C) decreased, and chlorophyll *a* and fucoxanthin increased in nutrient-rich conditions and varied between frond parts (Boderskov *et al.*, 2016). No distinct interaction of light and nutrients was determined. However, interactions of nutrients and light were found regarding sterolic compounds (de Jong *et al.*, 2021). Highest sterol content was measured at low nutrient and high light, although enhanced nutrient conditions combined with high light resulted in unchanged or even decreased concentrations. However, the authors attributed the results to reduced photosynthetic function rather than nutrient fluctuations.

A recent study on the interaction of nutrient availability and wave exposure revealed that fronds grow narrow under high wave exposure and in high nutrient concentrations and wider in low nutrient concentrations (Zhu *et al.*, 2021). Additionally, the biomass, shape and C:N ratio of the frond surface were affected by waves, nutrients and their interaction. Thereby, specific morphological changes can compensate for nutrient-poor conditions.

Eutrophication has become a common phenomenon in coastal regions, triggered mainly by anthropogenic nutrient input (Skjoldal, 1993; Norderhaug *et al.*, 2015). A moderately enhanced N (~3–20 µm) supply was reported to influence the physiology of *S. latissima* positively (e.g. Chapman *et al.*, 1978; Conolly and Drew, 1985; Gerard, 1997a). However, severe eutrophication levels combined with high temperatures are detrimental (Moy and Christie, 2012). In contrast, Arctic primary production was reported to be limited by low nutrient availability (<1 µm), but nutrient concentrations are expected to increase and alter seasonal patterns as melting, and thus freshwater run-off, increases and occurs earlier (Zacher et al., 2010; Filbee-Dexter *et al.*, 2019). Only marginal positive effects of nutrient enrichment on the physiological and biochemical status were reported in sporophytes of S. latisima in the Arctic (Gordillo, 2006; Diehl and Bischof, 2021). Temperature effects outcompeted nutrient supply, and no significant interactions of temperature and nutrients were determined (Diehl and Bischof, 2021).

*Saccharina latissima* can act as a bioremediator. In investigating the potential of *S. latissima* to remove nutrients from eutrophic brackish fjord systems and the parallel effects on several chemical compounds of the species, it was found to survive hyposalinity in elevated nutrient conditions (Mortensen, 2017). Higher protein and tissue N content and lower contents of β-glucans and iodine were found in young *S. latissima* maintained in brackish water with nutrient supplementation compared with conditions in seawater with adequate nutrient supply. Furthermore, the study revealed that the beneficial effects of increased nutrient levels were greater in young sporophytes than in older ones. The potential of algae to sequester nutrients poses great potential for establishing integrated multi-trophic aquaculture, which aims to reduce eutrophication caused by intensive fish farming (Kim *et al.*, 2015; Marinho *et al.*, 2015). While removing large amounts of N from the environment, *S. latissima* benefits from the elevated nutrient conditions by enhancing its growth by ≤50 % compared with a reference site (e.g. Sanderson *et al.*, 2012; Broch *et al.*, 2013; Wang *et al.*, 2014; Fossberg *et al.*, 2018). Different studies describe enhanced growth, photosynthetic activity, N (protein) concentration and pigment content, resulting in higher biomass quality of cultivated *S. latissima* (Sanderson *et al.*, 2012; Wang *et al.*, 2014; Rugiu *et al.*, 2021; for further information, see Saether *et al.*, 2023).

The effects of micronutrients on *S. latissima* are still largely unexplored. Trace metals are essential for various metabolic functions in seaweeds but can also be harmful at higher concentrations (Stengel *et al.*, 2005 and references therein). The only studies on the effects of microelements, e.g. iodine or copper, on *S. latissima* were conducted >30 years ago (Hsiao and Druehl, 1973; Brinkhuis and Chung, 1986; Chung and Brinkhuis, 1986). However, for other Laminariales, iodine has been shown to support osmotic functions (Nitschke and Stengel, 2014), iron had a strong impact on gametogenesis (Raymond and Stekoll, 2021), and copper modified the transcriptomic profile (Zhang *et al.*, 2019). The extent to which abiotic factors and distribution patterns affect the concentration of microelements in *S. latissima* is unknown. In addition, the fact that *S. latissima* accumulates micronutrients from the environment (e.g. Schiener *et al.*, 2015; Bruhn *et al.*, 2016; Nielsen *et al.*, 2016*b*) is of high relevance to the food industry, because concentrations above certain thresholds can exclude *S. latissima* biomass from human consumption (e.g. Bruhn *et al.*, 2019; Kim *et al.*, 2019; Roleda *et al.*, 2019).

### pH

Ocean acidification (OA) refers to the ongoing decrease in seawater pH and variations in carbonate chemistry resulting from the substantial marine uptake of CO_2_ since the Industrial Revolution (Doney *et al.*, 2020). Studies about the effects of OA on *S. latissima* have focused mainly on growth, photo-physiology and biochemistry. Ocean acidification has been reported to increase (Gordillo *et al.*, 2015; Olischläger *et al.*, 2017; Young and Doall, 2021), not affect (Iñiguez *et al.*, 2016; Olischläger *et al.*, 2017) or even decrease (Swanson and Fox, 2007) the growth rates of *S. latissima*, according to the duration of the experiment and the levels of *p*CO_2_ applied. Photophysiology, reflected by different parameters (e.g. pigments, photosynthetic O_2_ evolution and CO_2_ uptake, and chlorophyll *a* fluorescence), also showed various responses in OA conditions. For example, in some studies, it was shown that OA (~1000 and ~800 ppm, respectively) significantly increased the rates of photosynthetic CO_2_ uptake and O_2_ evolution rates (Longphuirt *et al.*, 2013; Nunes *et al.*, 2016), whereas another study failed to detect differences in net photosynthesis rates between ambient (390 ppm) and increased *p*CO_2_ levels (1200 ppm) (Iñiguez *et al.*, 2016). Regarding the biochemistry, *S. latissima* was found to use more CO_2_ than bicarbonate (HCO_3_^−^) as the photosynthetic carbon source, revealed by the signatures of a stable carbon isotope (δ^13^C) (Young and Doall, 2021). The contents of soluble carbohydrates, nitrogen and lipids changed in sporophytes of a temperate population of *S. latissima*, whereas they remained stable in the Arctic samples when *p*CO_2_ increased alone (Olischläger *et al.*, 2014). *Saccharina latissima* has been found to mitigate the negative effects of OA on farmed bivalves by increasing pH and the saturation state for aragonite (Young *et al.*, 2022). Thereby, the co-cultivation of bivalves and *S. latissima* is likely to be a promising integrated multi-trophic aquaculture approach to generate synergistic benefits in future OA scenarios.

The effects of OA on *S. latissima* have been investigated in interaction with temperature (Olischläger *et al.*, 2014, 2017; Iñiguez *et al.*, 2016) and UVR (Gordillo *et al.*, 2015). The effects of increased *p*CO_2_ on growth, biochemical composition and photosynthetic performances of *S. latissima* were generally less pronounced than those of increased temperature (Olischläger *et al.*, 2017). Furthermore, Arctic *S. latissima* was more resilient to increased *p*CO_2_ and more likely to benefit from climate change than the temperate population, as reflected by its increased growth rates at elevated *p*CO_2_ and higher temperatures (Olischläger *et al.*, 2014, 2017). The interactive effects of OA and UVR illustrated that OA increased the growth of *S. latissima*, meanwhile inhibiting a series of UVR-driven responses (e.g. pigments and photosynthetic electron transport) (Gordillo *et al.*, 2015). Owing to the various responses of *S. latissima* to OA discussed above, more work is needed to understand how OA is affecting *S. latissima* and will continue do so in the future. Besides, no studies on the molecular mechanisms regulating responses of *S. latissima* to OA are available to date, hence transcriptomics and/or metabolomics could help to understand the gene regulation and related metabolic pathways of *S. latissima* in OA conditions.

## BIOTIC INTERACTIONS

### Microbiome

Macroalgal functioning must be considered to be a result of the interactions between the algal hosts and their associated microbiota, forming a singular entity, the algal holobiont (Egan *et al.*, 2013). Algal microbial partners can be prokaryotes, such as viruses, Archaea or bacteria, and eukaryotes, such as fungi. Bacterial partners regulate and support macroalgal health and fitness (Goecke *et al.*, 2010), pathogen resistance (Wiese *et al.*, 2009), to a changing environment (Dittami *et al.*, 2016), and metabolism (Burgunter-Delamare *et al.*, 2020).

The *S. latissima* microbiota has become a subject of interest only in recent years (Vallet *et al.*, 2018; Tourneroche *et al.*, 2020; King *et al.*, 2022; Liu *et al.*, 2022; Burgunter-Delamare *et al.*, 2023). Bacteria associated with *S. latissima* are also found classically in other brown macroalgae (Hollants *et al.*, 2013) and belong predominantly to the Proteobacteria and Bacteroidota phyla (Tourneroche *et al.*, 2020; Burgunter-Delamare *et al.*, 2023). At the class level, Alphaproteobacteria and Gammaproteobacteria (Liu *et al.*, 2022; Burgunter-Delamare *et al.*, 2023), Deltaproteobacteria, Bacilli, Flavobacteriia, Planctomycetia and Verrucomicrobiae (Liu *et al.*, 2022) have been found. Bacterial strain isolation experiments determined that strains were affiliated with Actinobacteria, Bacteroidetes, Firmicutes and Alpha-, Beta- and Gammaproteobacteria and belonged to 21 genera (Wiese *et al.*, 2009). The genera *Marinobacter*, *Psychromonas*, *Litorimonas* and *Aquimarina* were also exclusively found attached to the blade of *S. latissima* and not in the surrounding seawater (Liu *et al.*, 2022). The bacterial composition changes gradually along the blade, shifting from a lower to higher alpha-diversity from the meristem to the distal part, reflecting the age gradient (Staufenberger *et al.*, 2008; Burgunter-Delamare *et al.*, 2022, 2023). The degree of colonization is linked, in part, to the types of metabolites released by the algae (Tourneroche *et al.*, 2020).

A bacterial core is found in *S. latissima* independent of the geographical origin, season or physiological state of the specimens. When looking at the meristematic part, a small core, comprising the four genera *Granulosicoccus* sp., *Litorimonas* sp., *Hellea* sp. and *Blastopirellula* sp., was found in two studies [8 of 13 Amplicon Sequence Variant (ASVs) and four of nine genera (King *et al.*, 2022); four genera (Burgunter-Delamare *et al.*, 2023)]. Five additional ASVs (*Croceitalea* sp., *Robiginitomaculum* sp., *Gammaproteobacteria* sp., OM190 sp. and KI89A_clade sp.) were also found in this blade region (King *et al.*, 2022). The bacterial core composition also shows shifts from low to higher diversity along the blade at the genus level. The distal bacterial core comprises the four genera found in the meristem core plus the five genera *Algitalea*, *Arenicella*, *Portibacter*, *Tenacibaculum* and *Bdellovibrio* (Burgunter-Delamare *et al.*, 2023). In addition, when looking at the core community and the ASVs found specifically attached to a particular tissue, particularly *Granulosicoccus* and *Litorimonas*, ecology and genome profiles suggest that they might be necessary functionally for the host (King *et al.*, 2022; Burgunter-Delamare *et al.*, 2023). For example, the Granulosicoccus genus might help its host thanks to key functions encoded in its genome (e.g. alginate metabolism, vitamin B_12_ biosynthesis, nitrogen reduction from nitrate to ammonium, or dissolved organic matter assimilation) and thus potentially providing the kelp with vitamins and available nitrogen (Kang *et al.*, 2018; Capistrant‐Fossa *et al.*, 2021; Weigel *et al.*, 2022).

Fungi infect the blade more often than other parts, and fungal communities comprise principally Ascomycota and Basidiomycota (Vallet *et al.*, 2018; Tourneroche *et al.*, 2020), with a predominance of Dothideomycetes and Sordariomycetes (Vallet *et al.*, 2018) or Psathyrellaceae (Tourneroche *et al.*, 2020). Additionally, *S. latissima* is colonized by viruses classified as Phaeovirus [*Saccharina latissima virus*, SlatV, family Phycodnaviridae (Schroeder and Mckeown, 2021)]. They are latent double-stranded DNA viruses that insert their genome into that of their host (McKeown *et al.*, 2017) and exist in three subgroups (A, B and C). Phaeoviruses are geographically widespread in the Laminariales (McKeown *et al.*, 2018). In particular, *Laminaria* and *Saccharina* genera are infected by Phaeovirus from subgroup C (McKeown *et al.*, 2017). Identifications of these viruses are supported by novel Phaeovirus major capsid protein (*mcpl* MCP) sequences found in kelp (by PCR) (McKeown *et al.*, 2017, 2018; Schroeder and Mckeown, 2021).

Environmental factors can influence the composition of the microbiota in *S. latissima* (King *et al.*, 2022). Several studies have compared the bacterial population from different geographical origins and found regional structuring in *S. latissima* [Baltic and North Sea (Staufenberger *et al.*, 2008; Lachnit *et al.*, 2009), North and West Scotland, Wales and South England (King *et al.*, 2022); Brittany, Helgoland and Skagerrak (Burgunter-Delamare *et al.*, 2023)]. The global epibacterial communities of *S. latissima* were differentiated between the Baltic and North Sea (Staufenberger *et al.*, 2008; Lachnit *et al.*, 2009). Differences regarding salinity, tidal range and bacterioplankton composition between sampling sites are likely to explain this. A regional structuring across British sites (North and West Scotland, Wales and South England) was also discovered, whereby bacterial communities in Wales differ from those in North and West Scotland. Here, the temperature is not the factor responsible, but rather the variable portion of the microbiota that reflects random and determinant processes within the host environment (King *et al.*, 2022), because reef habitats are highly dynamic and influenced by several factors that vary across multiple scales (Kaiser, 2011; Lamy *et al.*, 2018). In the same way, samples from Brittany, Helgoland and Skagerrak cluster according to their region of origin (Burgunter-Delamare *et al.*, 2023). Abiotic factors can lead to cellular stress and senescence and will thus create a new ecological niche for specific bacterial groups (Burgunter-Delamare *et al.*, 2023). Also, algal genotypes differ depending on the region (see ‘*Biogeographical patterns*’) (Guzinski *et al.*, 2016, 2020) and can impact bacterial communities. The chemical and lipid content in membranes also varies with environmental factors (see ‘*Responses to environmental drivers*’), hence attractiveness for bacteria is influenced (Burgunter-Delamare *et al.*, 2023). Furthermore, the associated microbial communities can vary with seasonality. Regardless of the mechanisms, seasonal changes can vary from site to site; therefore, any conclusions drawn about seasonality are valid only for the studied area. Differences between winter and spring were found at the blades and rhizoid levels of *S. latissima* from the Baltic Sea (Staufenberger *et al.*, 2008). In Brittany (Roscoff, France), the abundances of Firmicutes, Actinobacteria and Alpha- and Gammaproteobacteria were impacted, with an increase in autumn for the Firmicutes and Alphaproteobacteria, in summer for the Actinobacteria and in spring for the Gammaproteobacteria. The seasonal changes were linked to the nutrient content of seawater and the chemical composition of the algae (Burgunter-Delamare *et al.*, 2023).

Although the biological impact of viruses on their hosts is largely unknown, researchers are working on the microbial effects on the host regarding potential pathogens. By performing co-culture experiments with bacteria specifically isolated from *S. latissima*, it has been shown that a disruption in the microbiota composition (dysbiosis) is correlated with an increase in quorum sensing molecules (bacterial ability to detect and respond to cell population density through gene regulation) and a decrease in algal growth (Burgunter-Delamare, 2022). Also, *Aquimarina*, *Parcubacteria* and *Peronosporomycetes* were suggested as potential pathogens of *S. latissima* (Liu *et al.*, 2022). Conversely, initial evidence that fungal partners of brown macroalgae might protect their host *in vivo* by producing molecules as an active chemical defence has been provided by Vallet *et al.* (2018). Thus, the algal microbiota might manage the infection rate of pathogenic microbes in the phycosphere.

### Mobile biota

Kelps are essential coastal habitats for many commercially important fish and crustacean species (Seitz *et al.*, 2014). However, specific associations between fish/crustaceans and *S. latissima* have been poorly assessed. One study found 358 individuals of fish and crustaceans associated with *S. latissima* communities in Southern Norway, higher than the number of individuals associated with eelgrass and turf algae but lower than the specimens caught in forests of *Laminaria hyperborea* (700). Regarding species richness and diversity, eelgrass beds held higher diversity than *S. latissima* and the other habitats (Christie *et al.*, 2022). Habitat preferences of fish are species specific and vary with life stages. Young (<1-year-old) cod in Norwegian waters prefer red algae and eelgrass to habitats dominated by *S. latissima*, whereas cod >1 year old used all seaweed and seagrass habitats equally. In turn, the fishes Goldsinny wrasse (*Ctenolabrus rupestris*) and corkwing wrasse (*Symphodus melops*) preferred *S. latissima* and red algae over eelgrasses (Dunlop *et al.*, 2022). In the Northwest (NW) Atlantic, the residential fish cunner (*Tautogolabrus adspersus*) uses *S. latissima* and other large-blade Phaeophyta for foraging and refuge (O’Brien *et al.*, 2018). *Saccharina latissima* offers a better refuge for fish (>1 cm in length) but a lower-quality habitat for meso-invertebrates than other morphologically different macroalgae, such as turf (Ware *et al.*, 2019). However, a decline of large predatory fish has cascading effects throughout the food web, ultimately reinforcing the decline of *S. latissima* in some regions (Eriksson *et al.*, 2009).

### Epi- and endobiota

*Saccharina latissima*, like other kelps, can serve as a substratum, allowing smaller algae and animals to grow on (epiphytes) or inside (endophytes) its thalli (Bartsch *et al.*, 2008). Considering epiphytes, both macroalgae (e.g. *Ectocarpus siliculosus*, *Ulva lactuca* and *Champia parvula*) and microalgae (e.g. pennate diatoms, including genera *Licmophora*, *Navicula* and *Nitzschia*) were observed on the surface of *S. latissima* (Liu *et al.*, 2022). Considering endophytes, microscopic brown algae with filamentous thalli, mostly Ectocarpales *sensu lato*, are common in kelps (reviewed by Bartsch *et al.*, 2008) and in *S. latissima* (Bernard *et al.*, 2018). A study revealed that 88 % of endophyte algae from kelps belonged to the genera *Laminarionema* and *Laminariocolax*, with two isolates belonging to the genera *Ectocarpus* (Bernard *et al.*, 2019*b*). Furthermore, the most common endophyte in European *S. latissima* is *Laminarioema elsbetiae* (Bernard *et al.*, 2019*a*). The infection rates of endophytic algae in wild *S. latissima* along the European coasts were found to be ≤100 % (Bernard *et al.*, 2018). The occurrence and abundance of epi-/endophytic algae were affected both by environmental factors, such as seasons and locations, and by characteristics of *S. latissima*, such as age and position (Peteiro and Freire, 2013*a*; Bernard *et al.*, 2019*b*; Corrigan *et al.*, 2023). For example, the abundance of epiphytes on *S. latissima* was observed to be significantly higher for fronds growing in the sheltered area of the bay compared with those farmed at an exposed location, and the greatest quantities of epiphytes were on the apical parts of *S. latissima* blades (Peteiro and Freire, 2013*a*). Besides, cultivated *S. latissima* in Northern Brittany was not found to be affected by *Laminarioema elsbetiae*, which is highly prevalent in the wild populations of European *S. latissima* (Bernard *et al.*, 2019*a*). The infection with epibionts can reduce the photosynthesis of *S. latissima* by hindering ≤90 % of available light, revealed in laboratory conditions (Andersen *et al.*, 2018).

In addition to causing morphological changes, endophytic algae also adversely impact the physiological and biochemical traits of kelps, such as growth and reproduction. Transcriptomic analysis demonstrated that *S. latissima* upregulated many cell-wall modification-related genes and stress response-related genes during the infection of the endophyte *Laminarioema elsbetiae*, suggesting that endophytic algae damaged the cell wall and induced oxidative stresses in *S. latissima* (Xing *et al.*, 2021). In Norway, cultivated *S. latissima* sustains a heavy load of epibionts, ≤90 % of available area, causing light deprivation driven mainly by epiphytic algae and ascidians and, to a lesser extent, by bryozoans (Andersen *et al.*, 2018). The lack of *S. latissima* populations at the Skagerrak coast was suggested to be attributable to heavy epiphytism rather than the direct effect of abiotic factors on *S. latissima*, because transplanted sporophytes were able to grow and mature until the epiphyte load increased in the summer (Andersen *et al.*, 2011). The reduced growth and survival of kelp populations in shallow waters are also driven by the heavy load of epibionts, driving *S. latissima* populations deeper down and reducing their vertical distribution. This impact is seasonal and site specific; hence, it probably interacts with other environmental factors to drive the ongoing decline of *S. latissima* populations (Andersen *et al.*, 2018).

In the wild, the bryozoan *Membranipora membranacea*, which is an epiphyte on *S. latissima*, has negative effects on populations of *S. latissima* in the NW Atlantic, namely tissue weakening, breakage and, ultimately, loss of kelp biomass (Attridge *et al.*, 2022). Populations of this bryozoan, invasive in the Northeast (NE) Atlantic, are expected to increase under climate change scenarios, further impacting *S. latissima* populations in the area (Denley *et al.*, 2019). In the NE Atlantic, *M. membranacea* is a common native bryozoan, and although very little is known for natural populations, impacts of this species on cultivated *S. latissima* are already reported (e.g. Førde *et al.*, 2016; Forbord *et al.*, 2020). Another common bryozoan on kelps is *Electra pilosa*; however, this species has a slower growth rate and less substrate preference than *M. membranacea* and seems to have a more benign effect on kelps, including *S. latissima*; a pattern that holds on both sides of the Atlantic (Yorke and Metaxas, 2011; Førde *et al.*, 2016).

Mobile and epiphytic communities associated with *S. latissima* farms in Norway were shown to be significantly different from those associated with wild stands, holding less biodiversity and a smaller number of individuals (Bekkby *et al.*, 2023). The dominant species also differed between farmed and wild stands, with isopods being abundant in farmed *S. latissima* and nearly absent in the wild sporophytes. Also, kelp farms represent an additional, richer habitat than the surrounding water column (Bekkby *et al.*, 2023). An *S. latissima* farm in Sweden had a significantly positive impact on the amount and diversity of benthic infauna and attracted a similar number of mobile taxa to the nearby wild sites (Visch *et al.*, 2020). In a field study in Ireland comparing the associated biota of four macroalgae (*S. latissima*, *Halydris siliquosa*, *Fucus serratus* and *Sargassum muticum*), *S. latissima* held the lowest biomass of epiphytic algae of the four species (Strong *et al.*, 2009). *Saccharina latissima* supported a broad epiphytic faunal community (significantly different from the other macroalgae), with the species *Gibbula umbilicalis*, *Corophium volutator* and *Ischyrocarus anguipes* being characteristic of the thallus of *S. latissima*. In turn, the grazer amphipod *Dexamine spinosa* was considerably more abundant in *Sargassum muticum* than in *S. latissima* and had no significant effect on the growth of *S. latissima*. *Saccharina latissima* also showed more resilience to fouling (with only 9 % of biomass loss) when compared with the invasive *Sargassum muticum* (with mean losses of 70 %) (Strong *et al.*, 2009).

The biota associated with *S. latissima* in Kongsfjorden, a high Arctic fjord on the west coast of Spitsbergen, was assessed (Shunatova *et al.*, 2018). One hundred and eleven sessile taxa were reported in *S. latissima* individuals attached to stone in 2018: 80 animals (of these, 56 were Bryozoa) and 30 algae taxa (of these, 36 were Phaeophyceae and 11 Florideophyceae) (Shunatova *et al.*, 2018). Species richness associated with *S. latissima* was higher than in nearby sediment substrates. Both species richness and biomass varied with microhabitat and season, being considerably higher on the holdfast compared with blades and stipes and in January compared with May and September.

### Grazers

Although *S. latissima* contains high levels of phlorotannins that decrease the digestibility of the species, several animals can still graze directly on it. Among them is the snail *Lacuna vincta* (O’Brien and Scheibling, 2016; Young and Doall, 2021). A comparative study revealed that *S. latissima* is one of the preferred food sources for *L. vincta* and the macroalgae that elicits a higher growth rate of the snail (Chavanich and Harris, 2002). This snail prefers reproductive over vegetative tissue, probably owing to lower levels of phlorotannins in the former, compromising the reproductive success of *S. latissima* (O’Brien and Scheibling, 2016). *Lacuna vincta* also consumes *S. latissima* at higher rates when pretreated with high temperatures (21 °C), probably because the tissue is easier to consume (weaker and more fragile at higher temperatures) (Simonson *et al.*, 2015*a*). The grazing rate of *L. vincta* appeared to be unaffected by changing temperatures (Simonson *et al.*, 2015*a*) but decreased in OA conditions (Young and Doall, 2021).

A significant group in the coastal food web are sea urchins. Across the globe, events of mass grazing by sea urchins have decimated kelp forests and given rise to sea urchin barrens (Filbee-Dexter and Scheibling, 2014b). Several studies have shown that grazing pressure of the green sea urchin *Strongylocentrotus droebachiensis* led to the decline of *L. hyperborea* (e.g. Rinde *et al.*, 2014) in several areas in the NE Atlantic and of *Saccharina longicruris*, now *S. latissima*, in the NW Atlantic. Although field studies investigating the direct link between *S. droebachiensis* and *S. latissima* are rare, laboratory experiments show that *S. droebachiensis* indeed feeds on *S. latissima* (Daggett *et al.*, 2010; Eddy *et al.*, 2012), and growth rates of the sea urchins fed *S. latissima* or other species of macroalgae is similar (Carrier *et al.*, 2017).

The growth and survival of *S. droebachiensis* are, in turn, controlled by its predators (Norderhaug *et al.*, 2021) and by disease outbreaks (Feehan, 2014). A field and laboratory study in Nova Scotia showed that the presence of the crab *Cancer borealis* did not change the foraging behaviour of the sea urchin on *S. latissima*. A greater proportion of sea urchins around cages with *S. latissima* than without was also determined, revealing some response to a food cue (Harding and Scheibling, 2015). Another study revealed that juveniles of *S. droebachiensis* inhabiting *S. latissima* holdfasts are 20–30 % less likely to be predated by the crabs *C. borealis* and *Cancer irroratus* when compared with treatments with no refuge (Feehan *et al.*, 2019). Also, there was a correlation between *S. latissima* volume and the size of sea urchin juveniles, showing that *S. latissima* serves as food, habitat and refuge for *S. droebachiensis* (Feehan and Francis, 2014). Moreover, *S. latissima* detritus remains a main food source even for deep-living sea urchins (60 m) that can maintain a good reproductive status (Filbee-Dexter, 2014). In a laboratory experiment with samples of *S. latissima* from Alaska, a high sediment load (as in a land-terminating glacier) led to a sharp decrease in grazing rates of *S. droebachiensis* on *S. latissima*. In the same experiment, increasing temperature had no effect on grazing rates (Traiger, 2019).

Other species of sea urchin feed on *S. latissima*, such as *Arbacia punctulata*, although they prefer turf algae to *S. latissima* (Hamel, 2022). The purple sea urchin *Paracentrotus lividus* also feeds on *S. latissima* (Castilla-Gavilán *et al.*, 2019), although the best growth performance is achieved when fed on the red alga *Palmaria palmata*. A set of mesocosm experiments compared respiration and consumption rates of several grazers under medium and increased temperatures (Gilson *et al.*, 2021). The common sea urchin *Echinus esculentus* preferred the combination of *S. latissima* and *L. digitata* over *Laminaria ochroleuca* and *Saccorhiza polyschides*, the gastropod *Steromphala umbilicalis* consumed more of the latter, and the amphipod *Gammarus* spp. did not exhibit a preference. In addition, both *E. esculentus* and *Gammarus* spp. increased their respiration rates under warming, but only *Gammarus* spp. increased their consumption rates. In turn, *S. umbilicalis* increased growth with warming, but the other two species did not. Another animal group feeding on *S. latissima* are fish, such as wrasses, although *S. latissima* represents only a small percentage of their diet (Bourlat *et al.*, 2021). However, more studies looking at the gut content of fish are necessary to understand better the pressure exerted by this group of grazers.

A recent study revealed that kelp forests have recovered (*L. hyperborea* and *S. latissima* considered together) along the northern Norwegian coast (Christie *et al.*, 2019*b*). The recovery was suggested as the result of complex interactive effects of temperature on the food web. In the southern part of the previous sea urchin barren, the recovery of kelp is attributable to a decline in sea urchins following direct and indirect effects of increasing temperature (Christie *et al.*, 2019*b*), whereas in the northernmost regions of Norway, the recovery seems to be driven by top-down control. Overfishing of cod leads to a increase in predatory crustaceans, hence an decrease in sea urchin abundance, which results in a decreased grazing pressure on kelp (Christie *et al.*, 2019*b*; Norderhaug *et al.*, 2021). Given that this region is monitored closely (Moy and Christie, 2012; Christie *et al.*, 2019*a*, *b*), this could be an ideal opportunity to understand shifts between phases and determine what actions are successful in recovering *S. latissima* populations. Such knowledge can then be applied to less studied regions. Considering the diversity of animals feeding on *S. latissima* and the unknowns related to their interactions with other species and physical factors, more work is necessary to clarify the impact of grazing on *S. latissima*.

### Algal competitors

*Saccharina latissima* disappeared in the early 2000s from several sites in Norway and has been replaced by turf algae (Moy and Christie, 2012). Since then, several studies have tried to understand the underlying mechanisms and monitor any changes (e.g. Andersen *et al.*, 2018; Christie *et al.*, 2019*a*, *b*). Although some studies have reported that a regime shift has occurred (*S. latissima* was no longer able to recover and had been replaced by turf algae), recent monitoring efforts have revealed some recovery, although temporally and spatially variable. A similar regime shift has occurred in the NW Atlantic. Off Nova Scotia, Canada’s kelp biomass (mainly composed of *L. digitata* and *S. latissima*) was recently found to have decreased by 85–99 % when compared with the first monitoring campaigns in 1949 (Filbee-Dexter *et al.*, 2016). In the Gulf of Maine, a phase shift from canopy algae (including *S. latissima*) to ephemeral turf algae has occurred, and now 50–90 % of the bottom is dominated by red and green algae that were not common in the 1980s (Dijkstra *et al.*, 2017). Associated biota was found in lower numbers in *S. latissima* and other canopy species than in highly branched and filamentous algae. Nevertheless, high numbers of several gastropods were associated with *S. latissima*, including *Lacuna vincta*, *Margarite helicinus* and *Mitrella* (Dijkstra *et al.*, 2017). The presence of turf algae reduced *S. latissima* populations further by competing for space. *Saccharina latissima* is increasingly recruiting from turf algae, but the individuals are smaller, the survival rate lower, and they are more likely to be dislodged by wave action than sporophytes attached to rocky reefs, hence decreasing the health of the populations (Burek *et al.*, 2018; Feehan *et al.*, 2019). It was suggested that individuals are smaller because energy is diverted to larger holdfasts required to stabilize sporophytes in a more unstable substratum (turfs compared with rocks). Detachment rates of turf-attached *S. latissima* are more pronounced at high wave-action sites or after storm events. This pattern was consistent throughout the distributional range of *S. latissima* in the NW Atlantic.

A field study in Northern Ireland revealed that the invasive *Sargassum muticum* did not compete with *S. latissima* stands (Strong and Dring, 2011). Another potential competing species is the invasive green alga *Codium fragile* ssp. *fragile*. A study in Nova Scotia compared *C. fragile* with *S. latissima* in terms of the composition of its detritus and contribution to the detrital food chain (Krumhansl, 2012), revealing that degradation in *S. latissima* was faster and resulted in greater mass loss than *C. fragile*. The C:N ratio was higher in *S. latissima* than in *C. fragile* throughout decomposition, resulting in a lower nutritional value of *S. latissima* than of *C. fragile*. This resulted in associated macrofauna that was more abundant but less diverse on *S. latissima* than on *C. fragile*.

## BIOGEOGRAPHICAL PATTERNS

### Population differentiation at the genetic level

The population structure, genetic diversity and connectivity of populations of *S. latissima* have been explored in recent years (Guzinski *et al.*, 2016, 2020; Nielsen *et al.*, 2016*c*; Luttikhuizen *et al.*, 2018; Mooney *et al.*, 2018; Neiva *et al.*, 2018; Grant and Chenoweth, 2021). Overall, population differentiation, low within-population genetic diversity and low connectivity have been observed, although regional and local patterns can differ.

Only one study compared samples across oceans, identifying four differentiated phylogroups: (1) including specimens from NW Pacific (Japan, as *Saccharina coriacea*), NE (British Columbia) Pacific and Greenland and Hudson Bay in NW Atlantic; (2) NE Atlantic; (3) NW Atlantic; and (4) samples from Russia previously identified as *Saccharina cichorioides* (Neiva *et al.*, 2018). Together with recent findings on individuals in the NE Pacific and Bering Sea (Grant and Chenoweth, 2021), the hypothesis of a northern refugium during the Last Glacial Maximum for the species is gaining support, in contrast to the previous hypothesis of recolonization from southern European populations, as has been suggested for other seaweed species (Bringloe *et al.*, 2020). Further differentiation of *S. latissima* populations exists within the NE Atlantic phylogroup, with distinct ‘northern’ and ‘southern’ clusters (Neiva *et al.*, 2018). Those authors suggest that speciation might be in progress within these phylogroups, in accordance with another study determining population differentiation between seven European populations (Luttikhuizen *et al.*, 2018). Furthermore, it was shown that within-population genetic diversity is lowest for the southern populations (Spain and Portugal) and the isolated island population on Helgoland, German Bight, and highest in Spitsbergen (Guzinski *et al.*, 2016). This was also confirmed by a more recent study using both microsatellites and double digest restriction site-associated DNA sequencing, to explore the genetic diversity of 11 populations in the NE Atlantic (Guzinski *et al.*, 2020).

At smaller scales, populations of *S. latissima* revealed low genetic diversity within a brackish population (Denmark), while significant differences were observed between brackish and marine populations (Denmark vs. Norway and Sweden) (Nielsen *et al.*, 2016*c*). In the Irish Sea, populations from Scotland, the Isle of Man and Northern Ireland were also shown to be differentiated (Mooney *et al.*, 2018). In Norway, isolation-by-distance has been observed in *S. latissima*; however, the grouping seems to differ according to the method of analysis owing to the use of different genetic markers and sampling sites and sizes. In general, northern populations (Svalbard and Lofoten) are observed to be genetically distinct, suggesting that a physical barrier (islands) drives genetic differentiation. Overall, along the Norwegian coastline, results range from three different genetic groups (Evankow *et al.*, 2019) to generally connected populations (Ribeiro *et al.*, 2022). Local adaptation has been discussed for the general connection, because including a locus under positive selection altered the results of the genetic structure, even in the face of gene flow (Ribeiro *et al.*, 2022). Like European populations, a differentiation in ‘cold’ and ‘temperate’ clusters was found in the NW Atlantic phylogroup, although less pronounced (Neiva *et al.*, 2018). Fine-scale genetic structure and low within-population genetic diversity have been found for populations along the eastern Maine region in the NW Atlantic (Breton *et al.*, 2018). However, comparing the same markers, lower allelic richness and heterozygosity were reported in NW Atlantic than in NE populations (Guzinski *et al.*, 2016). Lower genetic diversity in the NW Atlantic compared with the NE has been reported for other benthic taxa (Wares and Cunningham, 2001). A recent study in *S. latissima* with more sampling sites revealed a biogeographical barrier at Cape Cod separating the populations in the Gulf of Maine and southern New England (Mao *et al.*, 2020).

Despite the apparent wealth of studies targeting the population structure of *S. latissima*, they differ in the locations studied and methods applied, preventing a wide comparison and global conclusions. All studies generally show that within-population genetic diversity is low, which is concerning because it indicates that populations might not have the adaptive potential to face increasing environmental change at sites where it is most extreme. Moreover, they report low connectivity that could result from stretches of land, waves and currents and variation in salinity depending on the site that restricts colonization of disturbed populations. For a successful conservation and/or restoration plan for the species, more data are needed on population differentiation, covering a large number of locations across the geographical distribution but also spatial heterogeneity at smaller scales (e.g. islands or other isolated populations). Different markers and sequencing depth provide slightly different results, which should be taken into account when choosing the methods.

However, most studies on population differentiation have neglected the epigenetic component of local adaptation, which is strong in *S. latissima* across latitudes (Scheschonk *et al.*, 2022). The epigenetic component might explain the general capacity of this species to adjust to rapid changes and colonize very different habitats. Hence, even with the apparent low genetic diversity, epigenetic differences might be high, and therefore it is crucial that they are considered in future studies.

### Phenotypic plasticity and local adaptation

Phenotypic plasticity refers to the ability of a single genotype to modify its phenotype in response to changing conditions (Nicotra *et al.*, 2010; King *et al.*, 2018). In contrast, ecotypes are locally adapted populations that are phenotypically and genetically differentiated for adaptive traits, meaning that they perform better in the local conditions than another population from a distant location with other local environmental factors (Kawecki and Ebert, 2004; Nicotra *et al.*, 2010). Ecotypes can emerge by long-term exposure to selective environmental pressures (Nicotra *et al.*, 2010), such as temperature ecotypes in different climate zones. For example, stress responses and recovery from ocean warming and heat waves were shown to differ between organisms and across latitudes (Winters *et al.*, 2011; Liesner *et al.*, 2020*a*). By local adaptation and acclimation mechanisms, species can vary in tolerance and performance to biotic and abiotic factors.

In models or simulations, broadly distributed species are usually treated as single homogeneous physiological units (Reed *et al.*, 2011). However, seaweeds such as *S. latissima* can exhibit different specific responses to distinct environmental conditions, of which temperature is a key factor (Lüning, 1990; Adey and Steneck, 2001; see also ‘*Responses to environmental drivers*’). Overall, the influences of various abiotic factors on the morphology, physiology and biochemical composition of *S. latissima* have been studied extensively, and a high degree of capacity for has been found. Little is known about how geographical patterns influence the capacity of the species.

Morphological plasticity is linked with adjustments to local conditions in different sites (Lüning, 1990; Peteiro and Freire, 2013*b*; Visch *et al.*, 2020; Zhu *et al.*, 2021; Diehl *et al.*, 2023). Effects of wave exposure on the frond length and width of *S. latissima* have been described in the field (Chapman, 1973) and in laboratory conditions (Gerard, 1987; Zhu *et al.*, 2021). Sporophytes typically form narrow blades with solid stipes in more wave-exposed habitats, whereas blades are broader with hollow stipes in sheltered habitats (Lüning, 1990). Specimens with hollow stipes will float when detached, possibly impacting the fate of detritus. Controlled laboratory experiments revealed an interaction between wave action and nutrient availability (Zhu *et al.*, 2021). Under wave action, *S. latissima* sporophytes developed a rough, more intricate frond surface that allowed for a higher nutrient and light uptake, resulting in high biomass and frond length even in low nutrient conditions (Zhu *et al.*, 2021). Additionally, sporophytes from a glacier-influenced area in Alaska have been described as narrower and longer than oceanic individuals (Spurkland and Iken, 2012), while in Svalbard (European Arctic), the biomass and size of *S. latissima* were lower in glacier-influenced sites. In the same fjord, sporophytes of *S. latissima* were longer and heavier at greater depths (Ronowicz *et al.*, 2022). For laboratory-grown individuals (from the gametophyte stage), sporophytes from the Arctic were narrower and longer than sporophytes from Brittany (Monteiro *et al.*, 2019*b*), indicating eco-phenotypes (see below). Morphological plasticity is very common in *S. latissima* and has led to misidentifications. For example, *S. angustissima*, formerly considered a morphotype of *S. latissima* (Augyte *et al.*, 2018), is endemic to Maine (USA). Very exposed conditions result in narrow blades; otherwise, it is morphologically very similar to *S. latissima* but shows genetic divergence.

Recent studies investigated the biochemical plasticity of field-grown sporophytes of *S. latissima*. By comparing the lipidomic composition and other parameters such as total carbon, lipid, protein, and carbohydrate contents of *S. latissima*, it was possible to distinguish populations from France, Norway and the UK (Monteiro *et al.*, 2020*b*). High intraspecific variability and habitat-specific phenotypes in morphology and biochemical composition were also found in field sporophytes of *S. latissima* across its entire distribution range in Europe, although without apparent geographical patterns (Diehl *et al.*, 2023).

In addition, different populations of *S. latissima* were shown to vary in sensitivity to environmental factors, such as temperature (Olischläger *et al.*, 2014, 2017; Monteiro *et al.*, 2019*b*; Diehl *et al.*, 2021, 2023). The existence of ecotypes regarding specific local parameters, such as temperature, salinity, *p*CO_2_ and light, have been postulated for the NE and NW Atlantic (Lüning and Dring, 1975; Gerard, 1987, 1988, 1990; Gerard and Du Bois, 1988; Müller *et al.*, 2008; Spurkland and Iken, 2012; Olischläger *et al.*, 2014, 2017). In contrast, other studies did not find evidence for ecotypic differentiation and instead suggested high phenotypic plasticity in *S. latissima* (Bolton and Lüning, 1982; Spurkland and Iken, 2011). Several studies have proposed ecological differentiation between populations from Spitsbergen and Helgoland (Müller *et al.*, 2008; Olischläger *et al.*, 2014, 2017). Differences in biochemical composition and physiological performance were reported under different temperature and CO_2_ treatments (Olischläger *et al.*, 2014, 2017). In a multiple-stressor experiment on laboratory cultures of *S. latissima* from Brittany and the Arctic, the results suggested the existence of ecotypes in *S. latissima* (Monteiro *et al.*, 2019*b*; Li *et al.*, 2020*a*). Responses to salinity and temperature variation diverged between Brittany and the Arctic, resulting in variations in morphology and in differences in growth rate, pigment content and gene-expression profiles. At the transcriptomic level, short-term responses differed between sporophytes from the two sites in magnitude and in the metabolic pathways involved, which were correlated to some degree with the local conditions (Monteiro *et al.*, 2019*b*).

Along the Norwegian coast (58–69°N), populations of cultivated *S. latissima* display higher blade length and biomass in central and northern regions that peak later in the season than for individuals in the south (Forbord *et al.*, 2020). Increased growth in north and central populations was coupled with higher protein content and delayed onset of biofouling.

Concerning vertical distribution, cultivated *S. latissima* sporophytes in Norway display higher biomass yields and frond length at 1–2 m depth compared with 8–9 m depth (Forbord *et al.*, 2020). However, this is not the case for the Baltic coast of Denmark, where frond size and dry matter reached the highest values at depths of >11 m (Nielsen *et al.*, 2016*b*).

To date, it has been shown that *S. latissima* is adapted to local conditions throughout its wide geographical distribution. Several studies focused on regional differences, however intra-regional, among-sites differences have also been shown (e.g. Smale and Moore, 2017; Wang *et al.*, 2021; Diehl *et al.*, 2023), which complicates the analysis of latitudinal effects on *S. latissima* but reveals its ability to acclimatize. Adjustments to abiotic drivers are site specific and, therefore, cannot be generalized from one population to the entire species complex. Nevertheless, definite ecotypes cannot yet be confirmed, and the question of whether *S. latissima* exhibits ecotypes or not is not fully resolved. In addition, most studies conducted on ecotypes so far have been focused on the genetic level as an explanation for the intraspecific variability (phenotypes as local expression of a genotype).

However, adaptation can also be powered by epigenetic mechanisms, which have been demonstrated recently in *S. latissima* (Scheschonk *et al.*, 2022). These findings show that, like the concept of phenotypic plasticity, the epigenome of *S. latissima* is likely to play a vital role in local and adaptation in this species. To highlight the importance of non-genetic gene control for local adaptation/processes, the term ‘eco-phenotype’ has been suggested (Scheschonk *et al.*, 2022). It indicates epigenetic mechanisms (within and across generations; see ‘*Epigenomics*’) to be involved in the variation of the phenotype in response to local parameters.

Phylogeographical differentiation of *S. latissima* populations has been reported across the Northern Hemisphere, also over small geographical distances (see ‘*Population differentiation at the genetic level*’). Although it is hypothesized that the European *S. latissima* species complex has not reached an equilibrium, the emergence of ecotypes could occur and eventually lead to different species (Luttikhuizen *et al.*, 2018; Neiva *et al.*, 2018). However, this might be precluded by the rapid changes in its habitats attributable to climate change. The fact that there is evidence that divergence between different populations is expressed at transcriptomic and epigenetic levels (Monteiro *et al.*, 2019*b*; Scheschonk *et al.*, 2022) suggests that ecotypes might emerge at the phenotypic level (or as more pronounced eco-phenotypes) in future or might be revealed with more extreme environmental pressure or testing of different parameters.

The variability in phenotypic plasticity and formation of ecotypes in *S. latissima* described above is based on different approaches (various laboratory experiments, *in situ* measurements and reciprocal transplants), environmental criteria (temperature, salinity and irradiance) and response parameters (growth, survival, fitness and biochemical composition). These differences complicate a systematic comparison of results and warrant a discussion of which parameters are most helpful in assessing phenotypic plasticity or local adaptation. ‘Common garden experiments’ or reciprocal transplants of field specimens from distinct populations are widely accepted methods to assess ecotypic differentiation (Kawecki and Ebert, 2004). However, reciprocal transplants cannot be applied in protected areas, such as Spitsbergen (Ministry of Climate and Environment Norway, 2001), and concerns regarding genetic contamination are warranted (Guzinski *et al.*, 2016; Luttikhuizen *et al.*, 2018). Hence, a combination of methodologies, both experimental work and omics tools, could provide a better picture of the existence of ecotypes in *S. latissima*.

### Ecological forecast

Climate change, especially global warming, has affected the distribution and abundance of many kelps (Smale, 2020; Fragkopoulou *et al.*, 2022). Kelps are projected to shift continuously northwards in the future (Wilson *et al.*, 2019; Krause-Jensen *et al.*, 2020). *Saccharina latissima* has already been observed and estimated to decrease in Nova Scotia (Filbee-Dexter *et al.*, 2016), the Gulf of Maine (Witman and Lamb, 2018), Rhode Island (Feehan *et al.*, 2019), Norway (Bekkby and Moy, 2011; Moy and Christie, 2012), Sweden (Eriksson *et al.*, 2002), Helgoland (Pehlke and Bartsch, 2008), the Iberian Coast (Casado-Amezúa *et al.*, 2019) and the eastern English Channel and Strait of Dover (Araújo *et al.*, 2016 and references therein), whereas it is increasing in biomass in Greenland (Krause-Jensen *et al.*, 2012, 2020) and Svalbard (Bartsch *et al.*, 2016) (see Fig. 1).

Species distribution models (SDMs) have been regarded as an effective tool for predicting marine species distribution shifts, using the species occurrence data and environmental variables available (Robinson *et al.*, 2011). In the last decade, SDMs have been applied to evaluate the distribution of *S. latissima* in Norway (Bekkby and Moy, 2011) and the British Isles (Yesson *et al.*, 2015). Furthermore, other models have considered the effect of climate change on the distribution of *S. latissima* and projected its future distribution trends (Müller *et al.*, 2009; Assis *et al.*, 2018; Goldsmit *et al.*, 2021). The northward shift of *S. latissima* was first projected by relating the temperature requirements of *S. latissima* and the modelling of sea surface temperature isotherms in 2080–2099 (Müller *et al.*, 2009). By constructing SDMs of kelp forests in the year 2100 under a future scenario (RCP 8.5), *S. latissima* was projected to extend to higher latitudes and inhabit the entire Arctic coast, while retreating from its southern limits in Nova Scotia, NW Iberia and Brittany towards Newfoundland and southwest Ireland (Assis *et al.*, 2018). In the Eastern Canadian Arctic, under RCP 8.5, *S. latissima* was projected to have the largest gain (64 000 km^2^) of suitable habitats in 2050 and second largest gain (17 000 km^2^) in 2100 of the kelps studied (Goldsmit *et al.*, 2021). However, some areas were projected to be lost in 2100, such as north of Baffin Bay, Foxe Basin and Hudson Bay (Goldsmit *et al.*, 2021).

Although SDM is a powerful tool to predict the potential distribution of species under future climate scenarios, the accuracy of predictions is often disputed. For example, few studies have taken into account in SDMs the physiological limits of seaweeds, although this has proved useful for modelling macroalgal distribution (Martínez *et al.*, 2015). Besides, the discrepancy between model predictions and long-term field observations of the abundance of Arctic kelps suggests that SDMs might overestimate the potential of kelps for northern expansion in the short term (Filbee-Dexter *et al.*, 2019). The possible reasons might be the extensive gaps between available substrates, the limited dispersal ability of kelps, and other abiotic factors, such as turbidity and light penetration (Filbee-Dexter *et al.*, 2019; Smale, 2020). Hence, it is crucial to track the occurrence and absence of *S. latissima* throughout the whole distributional limit in the future to improve the precision of model predictions. Modelling exercises that include physiological data generated from experiments and that account for possible local adaptation are also worth considering. To achieve more accurate predictions, it is also essential to improve the spatial resolution of environmental data layers available to consider the variable physical landscape of the intertidal and shallow subtidal zones where *S. latissima* occurs and to account for regional patterns that might override large-scale warming patterns, e.g. upwelling (Potter *et al.*, 2013; Meneghesso *et al.*, 2020).

## CONSERVATION AND RESTORATION

Given the severe decline of kelp forests globally, action is needed to protect these important ecosystems in the future. Threats to *S. latissima* have been discussed in previous sections (effects of abiotic and biotic factors largely driven by climate change). Evidence of the impacts of other anthropogenic activities, such as pollution, on *S. latissima* is scarce. The rare examples include hydrogen peroxide on salmon farms that induced significant mortality and reduced photosynthetic efficiency of nearby *S. latissima* juveniles (Haugland *et al.*, 2019). In contrast, *S. latissima* juveniles at sites impacted by the Exxon Valdez oil spill presented higher densities than reference sites 2 years after the spill, and populations recovered 10 years later (Dean and Jewett, 2001).

Kelp forests have been included in conventions aiming to protect habitats, namely the Convention of Bern and the Habitats Directive, both at the European level, and in the list of threatened species and habitats of the Convention for the Protection of the Marine Environment of the NE Atlantic (OSPAR) (de Bettignies *et al.*, 2021). Nevertheless, specific measures targeting conservation of kelps and, more specifically, *S. latissima* are rare. Marine Protected Areas (MPAs) in the Atlantic have not yet been designed to protect kelp forests, but many include areas with kelp forests, providing some protection because harvest is forbidden. This is the case in some MPAs in Norway, France, the UK and Germany. However, the effects of these measures have not been evaluated, and little is known about the efficiency of MPAs in conserving kelps (de Bettignies *et al.*, 2021). A study in California, USA, revealed that after 15 years, the abundance of sea urchins inside the MPA remained unchanged and giant kelp populations did not differ between inside and outside the MPA (Malakhoff and Miller, 2021). However, another study in a 30-year-old marine reserve in New Zealand demonstrated that the MPA effectively conserves populations of the kelp *Ecklonia radiata*. Outside MPAs, where fishing still occurred, sites were dominated by sea urchins and turf algae, whereas inside the MPA, healthy populations of *E. radiata* were present (Peleg *et al.*, 2023). Marine Protected Areas in Chile have successfully preserved intertidal populations of the commercially harvested *Lessonia* spp. (González-Roca *et al.*, 2021). These are encouraging results and call for similar actions for *S. latissima* if aiming for the protection and/or restoration of its populations. Considerable baseline information will be required to evaluate the effect of MPAs and other conservation measures, such as reducing local pollution inputs or limiting coastal construction, on the conservation of *S. latissima*.

If conservation actions fail, restoration might be the way to go. One strategy to recover populations is to plant new individuals where they have been lost/decreased, aiming to restore the populations. A few studies aiming to find the best techniques for restoration have been performed on *S. latissima* (Fredriksen *et al.*, 2020; Tsiamis *et al.*, 2020; Le François *et al.*, 2023). In a trial in Quebec, Canada, the production of *S. latissima* sporophytes was successful and worked best on artificial substrate and using a binder-based method for spraying gametophytes (Le François *et al.*, 2023). In contrast, a study in Scotland revealed that the abundance of *S. latissima* and other kelps in an artificial reef was low, and in turn, turf seaweeds were abundant (Tsiamis *et al.*, 2020). This is in accordance with a review on artificial seaweed reefs that concluded that the success of reforesting macroalgae is variable and depends on the scale, structural composition, materials used and surface complexity (Jung *et al.*, 2022). A trial in Norway was also successful using the ‘green gravel’ method, in which stones are seeded in the laboratory and are planted in the field only when sporophytes reach 2–3 cm (Fredriksen *et al.*, 2020). Another strategy for restoration of kelps is grazer control. A study in Norway showed that sea urchin decline following treatment with quicklime allowed for kelp forest recovery, including *S. latissima* (Strand *et al.*, 2020). Other strategies not yet tested for *S. latissima* include the harvest of grazers and destructive hammering of sea urchin populations (Eger *et al.*, 2022). Up to now, research on restoration practices in *S. latissima* is scarce, and no large-scale restoration plan has been attempted.

Scientific debate is ongoing on whether assisted evolution (or assisted adaptation) is warranted when restoring degraded and vulnerable populations. Assisted evolution entails that the genetic diversity of populations is increased artificially, by moving new genotypes to a population, boosting genetic diversity within, using intraspecific hybrid vigour or heterosis or genome editing (Coleman *et al.*, 2020; van Oppen and Coleman, 2022). These methods raise important ethical questions that might limit their use (Filbee-Dexter and Smajdor, 2019). Overall, this is an area of research that we expect to attract a lot of attention in the near future as the need to restore degraded habitats becomes evident, and best practices need to be discussed.

## CONCLUSIONS

**Fig. 4.**
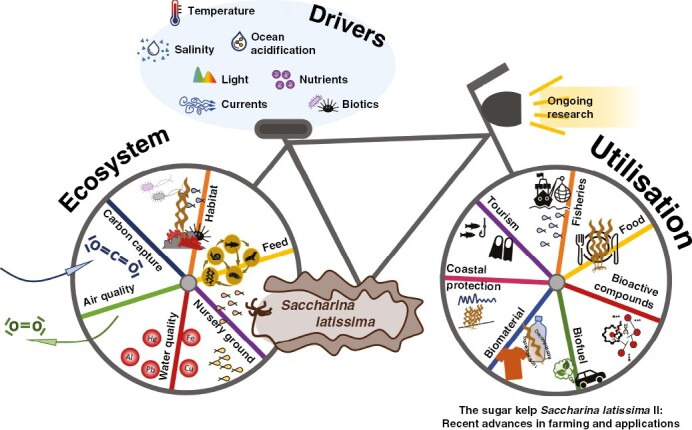
Research values of *Saccharina latissima* sporophytes: ecosystem services, economic values and drivers. Schematic display of the manifold ecosystem services and economic application. *Saccharina latissima* is represented as a bicycle chain powering many ecosystem services: providing habitat, feed and a nursery ground for the associated micro- and macrofauna (see main text, section ‘*Biotic interactions*’); improving the water quality by accumulating high concentrations of harmful elements; improving the air quality by releasing oxygen; and sequestering carbon. These ecological values lead to a multitude of economic values. In nature, *S. latissima* provides coastal protection by reducing wave energy, increasing fishing and diving tourism, and enhancing fisheries by serving as a nursery ground for economically important fish species (‘*Biotic interactions*’). Harvested *S. latissima* is used for: food; feed; extraction of bioactive compounds, with applications in pharmaceutical, medical, cosmetics, paper and processed food industries, among others (see more in Saether *et al.*, 2023); and development of biofuels and biomaterials (see more in Saether *et al.*, 2023). The main drivers of *S. latissima* survival and growth are temperature, light availability, salinity, nutrients (see ‘Response to environmental drivers’) and biotic factors (‘*Biotic interactions*’) that significantly modify the ecological and economic services provided. Ongoing research leads the way for a deeper understanding of kelp ecosystems and new applications (‘*Conclusion*’).

All in all, *S. latissima* has been studied intensively over the last 15 years, and important new insights have been gained (Fig. 4). Nevertheless, new findings usually raise new questions, and here we highlight the most current research priorities.

Generally, as already stated in the review of the genus *Laminaria* by Bartsch *et al.* (2008), microscopic life-history phases have received considerably less research attention than the sporophyte stage. Spores, stages of gametophyte development, gametes and microscopic sporophytes should all be studied more intensely. Direct comparisons between life-history stages have to be included in future studies to identify phase-specific responses to environmental drivers. Knowledge is lacking on demographic patterns, life span and the spatial and temporal variability of life-cycle stages. Also, studies on differences in gametophyte sexes and sporophyte maturity are largely underrepresented. Only by examining the sensitivity throughout the entire life cycle and across the geographical distribution of *S. latissima* will it be possible to gain a comprehensive understanding of the resilience of the species to climate change, which is an important component for management of conservation and cultivation. Regarding climate change, most attention has been given to the impact of warming and marine heat waves. However, other weather extremes, such as marine cold spells (Schlegel *et al.*, 2021) or climate change-related increases in storm surges, can have a huge impact and should be considered in future studies. Furthermore, to date, studies investigating the impact of irradiation on *S. latissima* have focused mainly on changes in PAR and the effect of UVR. However, increased sediment input along all coastal regions (meltwater run-off, river outflows and precipitation) not only leads to a reduction of PAR but also affects the spectral composition of the water column. Especially in Arctic regions, the environmental light spectrum changes drastically owing to accelerating glacial melt and permafrost thaw, reducing the photosynthetically available radiation (Niedzwiedz and Bischof, 2023). Therefore, in further experimental and modelling research on *S. latissima*, the spectral composition of radiation should be incorporated.

The strongest impact of climate change on marine life has been observed in the Arctic (Masson-Delmotte *et al.*, 2021), where pronounced seasonal light conditions exist. Overall, seaweeds in Arctic regions have been studied intensively (Lebrun *et al.*, 2022). Nevertheless, the adaptive responses to polar day, polar night and the respective transitions are poorly investigated. Furthermore, melting sea ice and glaciers change salinity or result in coastal darkening (Konik *et al.*, 2021), which can result in additional stress for Arctic *S. latissima* and should be analysed further. In addition, increasing temperatures are especially pronounced during Arctic winters, with significant environmental consequences (Maturilli *et al.*, 2015). However, only very few winter data for Arctic *S. latissima* are available. In this context, transgenerational effects in cold have been shown for *L. digitata* (Liesner *et al.*, 2020*b*), and the same might hold for *S. latissima*. Data on growth rates, stress responses and biotic interactions for the rear-edge populations of *S. latissima* are also lacking. The uneven distribution of studies across the distributional range of the species (focusing on central populations in Germany, the UK and mainland Norway) limits our understanding of its potential for to various environmental conditions. To date, the question of whether *S. latissima* exhibits different ecotypes remains unanswered and requires further research.

When testing the consequences of climate change, an important and very complex topic is the interaction of drivers. Hence, multifactorial approaches are being applied increasingly but are still a minority, despite their high ecological relevance. The interplay of various altering factors might have synergistic or antagonistic impacts on the resilience and susceptibility of *S. latissima*, hence these factors are key to understanding survival and success in the future. Experiments testing the impact of ongoing climate change mostly use average values over large scales, e.g. average sea surface temperature increase, and fail to include relevant temporal and spatial variability at different scales (Seabra *et al.*, 2015; Bates *et al.*, 2018). Different intensities, durations and recovery periods in marine heatwave experiments result in different responses of *S. latissima*. Moreover, inter-annual and seasonal variability in the thermal stresses of *S. latissima* has been shown (Niedzwiedz *et al.*, 2022). In general, seasonality strongly impacts the physiological and biochemical parameters of *S. latissima*; however, little is known about how phenology changes across the distributional range and how it is affected by climate change. Future research needs to include more intricate experimental designs that address more variability and how it might affect the survival of *S. latissima*.

The application of ‘omics’ to *S. latissima* is expected soon to increase sharply, as costs decrease and technologies quickly improve. Nonetheless, ‘omics’ approaches to *S. latissima* and other kelps lag behind other major taxonomic groups, and there is still much to be explored. Recent work on the transcriptomic responses in *S. latissima* should be expanded to include more abiotic and biotic drivers and complex interactive responses to climate change. In addition, transcriptomic studies should be combined with metabolomics and proteomics to understand how regulation occurs fully. However, a major caveat to these approaches is the lack of functional annotation, which limits our interpretation of results. More efforts in the molecular and biochemical characterization of genes are necessary, and knowledge generated for *S. japonica* (a closely related species) will help to streamline progress for *S. latissima* (e.g. Zhang *et al.*, 2018b).

Another severe knowledge gap is how epigenetic mechanisms modulate responses in *S. latissima*. The modulation of DNA methylation in response to an environmental stimulus has recently been demonstrated in *S. latissima* (Scheschonk *et al.*, 2022), but whether non-coding RNAs and histone modifications are also involved has not yet been tested. Given that these last two mechanisms have been demonstrated in other brown algae (Bourdareau *et al.*, 2021; Bai *et al.*, 2023), studies examining these patterns in *S. latissima* will surely follow. In addition, active gene modulation would be required to assess the definite impact of any given epigenetic modulation on the gene expression.

Regarding the microbiome, most microbiota studies for *S. latissima* have focused on describing the microbial partners. Consequently, there is a need to expand the research on co-cultures to investigate causal relationships. Specific isolates of interest, such as bacterial core, specialized metabolizers and pathogens, can be used to study their impact on algal growth and morphology (Burgunter-Delamare, 2022). Furthermore, more research is needed on the impact of potential pathogens on the physiological state of *S. latissima* and the composition of its entire microbiota. *In silico* predictions of beneficial metabolic network complementarity are a way to identify specific interactions between *S. latissima* and its microbiota. There is also a need to start cataloguing genes and their functions for both the microbiome and the host, which will require a combination of metagenomic and metatranscriptomic studies linking microbial and host gene expression. Viruses have been described recently in Laminariales and reported to infect two-thirds of the host populations (McKeown *et al.*, 2017), highlighting the importance of incorporating viruses in studies on algal microbiota.

All the ‘omic’ data recently generated are being used to improve breeding of macroalgae, which still lags far behind plant crops. Several of these land crop techniques are expected to be applied to *S. latissima* as investment in aquaculture facilities is rising on both sides of the North Atlantic. However, these techniques might raise social and ethical issues that will need to be discussed with society in the next decades (for a discussion on the topic, see Charrier *et al.*, 2020).

Although the distribution of *S. latissima* is fairly well documented in some regions, repeated monitoring and detailed distribution data are lacking in other regions, e.g. south of Europe and Russian waters. New technologies, such as remote sensing, drone imagery, video by underwater vehicles and environmental DNA approaches can assist greatly in monitoring the occurrence of *S. latissima* (e.g. De Pooter *et al.*, 2017; Douay *et al.*, 2022). Studies across the biogeographical distribution range of *S. latissima* will help to distinguish between present phenotypic plasticity and adaptation patterns present in the species and how it might be affected by climate change scenarios.

Despite overwhelming evidence that *S. latissima* populations are declining and that this compromises the ecosystem services they provide, there are still few management actions in place. Moreover, if present, these are country or region specific, without international perspective and guidance. Hence, the effectiveness of management actions already applied to other macroalgae has not been tested for *S. latissima*. It is imperative that this is put into action if we aim to maintain the remaining populations and restore some of the others. Management actions tested in other seaweeds that might also prove successful with *S. latissima* include improving water quality (by decreasing nutrient load, for example), Marine Protected Areas and grazer control (Strain *et al.*, 2015; Eger *et al.*, 2022; Peleg *et al.*, 2023). As political interest and societal benefits in recovering kelp populations are increasing, securing the financial and logistical means to undergo large-scale restoration efforts might become more feasible (Eger *et al.*, 2020; Filbee-Dexter *et al.*, 2022*c*).

## Data Availability

This article has been submitted to the preprint server EcoEvoRxiv (https://doi.org/10.32942/X2W59T).

## Notes

Dedicated to Christian Wiencke on the occasion of his 75th birthday
